# Estimating the cost and affordability of healthy diets: How much do methods matter?

**DOI:** 10.1016/j.foodpol.2024.102654

**Published:** 2024-07

**Authors:** Derek Headey, Kalle Hirvonen, Harold Alderman

**Affiliations:** a The International Food Policy Research Institute (IFPRI), United States; b United Nations University World Institute for Development Economics Research (UNU-WIDER), Finland

**Keywords:** Food prices, Healthy diets, Nutrition, Poverty, International Comparison Program (ICP)

## Abstract

Recently developed cost and affordability of healthy diet (CoAHD) metrics have quickly become mainstream food security indicators. However, published research on the sensitivity of estimation methods is limited. This paper focuses on two important innovations in CoAHD measurement at the global level. First, we develop a demographic scaling factor to adjust healthy diet costs for cross-country differences in age structures, since younger populations generally require fewer calories than older populations. Second, we improve the way in which household expenditure available for purchasing food (“food budgets”) are derived. In addition, we explore sensitivity of global CoAHD estimates to potential problems with the representativeness and food product coverage of global food price data and vary assumptions for activity levels that shape energy expenditure requirements. We apply these explorations to the EAT-*Lancet* reference diet in 137 countries using price data from 2017. Relative to the conventional methods, we find that demographic scaling and improved food budget derivation substantially reduces the estimated population who cannot afford a healthy diet, from 3.02 to 2.13 billion. Adjustments for low product coverage can lead to modest reductions for specific regions and food groups, while higher physical activity assumptions increase the share of people who cannot afford a healthy diet, though perhaps implausibly so. Methods clearly matter in CoAHD estimation, and more accurate and timelier CoAHD estimates have substantial scope to improve policy analysis, design and targeting.

## Highlights

• Cost and affordability of healthy diet (CoAHD) are now mainstream food security metrics.
• We explore innovations and extensions for improving global CoAHD estimates.
• Demographic differences across countries have sizable impacts on CoAHD estimates.
• CoAHD metrics also very sensitive to assumptions on available income for food budgets.

## 1 Introduction

Poor diets are now the leading behavioral risk factor in the global burden of disease (Gakidou et al., 2017). Although there are both economic and non-economic barriers to improving diets and food security (Barrett, 2010), research on economic barriers was – for many decades – mainly focused on the costliness of healthy food for poor people in rich countries (Darmon and Drewnowski, 2015, Drewnowski and Darmon, 2005, Drewnowski and Specter, 2004). Yet it is self-evident that food affordability is a far more binding constraint on improving diets in low- and middle-income countries (LMICs). Nevertheless, only recently have researchers developed methods for documenting these constraints by estimating the cost and affordability of healthy diets (CoAHD) as defined by food-based dietary guidelines (Bai et al., 2020a, Dizon et al., 2019, Headey et al., 2023, Herforth et al., 2020, Hirvonen et al., 2020, Mahrt et al., 2019, Raghunathan et al., 2021). In a short space of time, this research has been exceptionally influential, prompting international institutions like the Food and Agriculture Organization (FAO), the World Food Programme (WFP) and the World Bank to publicly adopt CoAHD metrics, notably in the UN’s State of Food Insecurity and Nutrition (SOFI) annual reports, which conclude that around 3 billion people cannot afford a healthy diet (FAO et al., 2022). The FAO is also encouraging national and regional CoAHD measurement and monitoring, while the *Food Prices for Nutrition* project has been assisting governments in Nigeria (NBS, GAIN, 2023) and Ethiopia (Alemayehu et al., 2023) to develop their own CoAHD metrics and reports using national data and national food-based dietary guidelines. Moreover, both the FAO (2023) and The World Bank (2023) now report country-level CoAHD metrics on their widely used global databases, thus greatly improving the accessibility of these metrics for monitoring and research purposes.

Although the addition of CoAHD metrics to the suite of global food security indicators is a welcome outcome, the speed at which these metrics have been mainstreamed has resulted in limited sensitivity analyses regarding the various assumptions that underlie these estimates. Measuring CoAHD at a global level, requires a number of decisions on operationalizing dietary guidelines, potentially strong assumptions on the representativeness of international food price data, and imputations to estimate what we term “food budgets” (the income or expenditure that a household has available for spending on a healthy diet).

In this study our objective is to explore the extent to which decisions over such methods matter in the estimation of CoAHD metrics, with a focus on two innovations and two sensitivity tests that we hypothesized, *a priori*, to be both quantitatively important in influencing global CoAHD estimates, and feasible enough to practically pursue.

The first innovation is to adjust CoAHD estimates for the sizable demographic differences across populations. Existing CoAHD approaches specify a balanced and diverse healthy diet with caloric requirements for a hypothetical 30-year female adult who is neither pregnant nor lactating (FAO, IFAD, UNICEF, WFP, WHO, , 2020, Herforth et al., 2020, Hirvonen et al., 2020). However, daily energy requirements vary by age and sex, with younger children having much lower energy requirements than adults. We therefore hypothesized that the considerable demographic differences across countries (World Bank, 2016) could mean that healthy diet costs are over-estimated for LMICs with younger populations. We correct this bias with a novel demographic scaling factor.

The second innovation pertains to the derivation of the share of household income or expenditure available for spending on food, or more specifically, for spending on a healthy diet food basket. How to do so is not obvious or straightforward, posing an important challenge for global CoAHD estimates. Hirvonen et al. (2020) abstracted away from this altogether by comparing the EAT-*Lancet* diet costs to total incomes and reporting the percentage the population who cannot afford the diet even if they spent their entire income on food. National CoAHD analyses typically compare healthy diet costs to actual food expenditures from national income or expenditure surveys (e.g., Mahrt et al., 2019). To overcome this problem at the global level, the 2020 SOFI report (FAO et al., 2020) assumed that households need to spend at least 63 percent of their income/expenditure on food (37 percent non-food expenditure allowance), irrespective of their income levels, based on the observed share of income the poorest quintile of people in lower income countries (LICs) spend on food. In 2022 this was revised to 48 percent (52 percent for food) and again applied across the board (FAO, Ifad, UNICEF, WFP, WHO, 2022). Yet the application of common food and non-food expenditure shares irrespective of income levels is problematic since the cost of certain non-food basic needs increase with country income levels (e.g., housing), but also if environmental factors play a role in influencing essential non-food expenditure requirements (Allen, 2017); for example, populations in colder climates have higher costs for clothing and heating. We therefore suggest an alternative means of deriving food budgets, by extending a method developed by Allen (2017) to systematically predict non-food expenditure requirements across countries and derive food budgets as the difference between total income/expenditure and non-food income/expenditure.

While we hypothesized that demographic adjustments and revisions to food budget estimation could have substantive impacts on CoAHD estimates, we also conducted two additional sensitivity tests that could potentially reveal other empirical concerns with CoAHD estimation.

First, in the global context, CoAHD estimates have used either the 2011 or 2017 International Comparison Program (ICP) surveys of retail prices for standardized food products (World Bank, 2020). While the availability of this exceptionally rich data has catalyzed a wide range of innovative research on food prices and diets (Allen, 2017, Bai et al., 2020a, Headey and Alderman, 2019, Hirvonen et al., 2020), there are justified concerns about the representativeness of ICP data for CoAHD estimation purposes. As ICP documents acknowledge (World Bank, 2020), consumer price surveys are potentially vulnerable to urban bias in more rural LMIC populations, and urban food prices will often systematically differ from rural prices.^1^ Another concern is that some ICP countries have shorter food product lists than others, most likely due to artifactual reasons rather than any genuine differences in dietary or market diversity. Short product lists risk excluding low-cost foods that might otherwise appear in least-cost healthy diets, thereby potentially inflating CoAHD estimates. We therefore perform a sensitivity check to assess the degree to which these potential limitations in the ICP price data may affect CoAHD estimates.

Second, earlier CoAHD analyses assume uniform physical activity levels across countries and therefore common calorie requirements, even though more arduous manual labor – such as non-mechanized farming and household chores such as collecting water and fuel– is much more common in LMICs than it is in high income countries. Recent research integrating data on energy expenditure from accelerometers with time-use data shows that reductions in physical labor can have large effects on human energy (calorie) requirements, with an hour of drudgery reduction reducing energy requirements by 11–22 percent for men and 13–17 percent for women in Ghana and India (Srinivasan et al., 2020). The empirical challenge is that no such data is available in national surveys, so developing precise country-specific energy requirements based on occupational structures is beyond the scope of this paper. Hence, we explore this issue somewhat simplistically by conducting a sensitivity check to evaluate the impact of varying assumptions regarding activity levels on the CoAHD estimates.

Finally, we conclude the paper with a discussion of key findings, key limitations of our analysis, and possible extensions for future research on CoAHD measurement. If CoAHD metrics are indeed to be used for regular monitoring of food security as well as policy analysis (FAO et al., 2022), then it behooves researchers to transparently demonstrate how much methods matter, and to improve the rigor of CoAHD measurement to the maximum extent possible.

## 2 Data and methods

We use the most recent ICP data to carry out the two innovations and perform the two sensitivity checks introduced in the previous section. In this section, we provide the details on the data and methods we use.

### 2.1 The data: The International Comparison Program (ICP) database

The key database underlying global CoAHD estimates is the ICP retail food price dataset. The broad steps involved in the collection, compilation, and construction of these averages are outlined in ICP reports (World Bank, 2013, World Bank, 2020), though the exact processes of data collection for each country can vary. First, the ICP Global Office and the regional implementing agencies (e.g., the African Development Bank) set out an aspirational list of Standard Product Definitions (SDPs) of food products defining commodity type, variety, quality, quantity, packaging or even suggested example brand names, that should ideally be priced in as many countries as possible. A regional list is sent to national implementing agencies (usually national statistical agencies) for price collection, although there is large variation across countries in the number of food items priced, as we show below.^2^ ICP food products are not chosen for any nutritional basis, but according to two standard economic criteria: *representativeness* of foods actually consumed in a country and *comparability* of products across countries.^3^ However, the ICP explicitly notes that “there is a tension between the two criteria, *representativeness* and *comparability*, and so the ICP strives to strike a balance between these two requirements” (World Bank, 2013, p. 27). Ethiopia, a country of over 100 million people, provides examples of this tension. Among the most important staples in the country are *teff* and *enset* (false banana). Although these two foods account for about 19 percent of the of calories consumed in Ethiopia (Stifel and Hassen, 2020) – and could conceivably be cheap enough to frequently appear in a least-cost nutritious diet – neither appear in the ICP food list because they are essentially only consumed in Ethiopia, thus failing the comparability criteria. Hence it is important to bear this limitation in mind when using ICP data for least-cost diet comparisons across countries.

After price surveys are conducted in the participating ICP countries, individual price quotations for a product are used to construct *national annual average prices* from a range of outlets, including supermarkets, local stores and markets, and vendors spread – to varying degrees – across areas within a country (rural and urban and subnational units). The national average prices usually entail a self-weighting design in which price quotes are spread across outlets and regions broadly in proportion to their importance (sales or quantities) in the economy, on the assumption that national implementing agencies already strive to collect nationally representative consumer price data. In practice, though, all price surveys in highly rural economies suffer from some degree of urban skew as collecting price in remote rural communities is very costly.^4^ This need not necessarily result in any sizable bias, but the challenge of achieving spatial representativeness in national price surveys should not be taken lightly. Indeed, ICP analysts often engage in an explicit re-weighting of price observations to correct for urban bias if sufficient geographical information is available (World Bank, 2013). However, in cases in which no weights were available, simple arithmetic averages of the prices are used.^5^

### 2.2 The diet: Re-estimating EAT-Lancet reference diet (ELD) costs and affordability

To investigate the sensitivity of CoAHD methods we use the EAT-*Lancet* reference diet (ELD) as an example because it is intended to be a global reference diet, although our results would also extend to the healthy diet used in the most recent SOFI reports (Herforth et al., 2022). The ELD is based on a balanced diet calibrated to a 2,503 kcal per person per day diet, corresponding to the average energy needs of a 30-year-old woman weighing 60 kg and whose physical activity level is between moderate and high (Willett et al., 2019). The specific serving sizes for each food group are derived from available scientific evidence about health risks, although one study shows that the ELD could result in inadequate micronutrient intake (Beal, Ortenzi and Fanzo, 2023).^6^ In Table A1 in the Appendix A we show the 21 more detailed food groups as well as the 15 “functional” food groups used in this analysis and in Hirvonen et al. (2020). The ELD recommended a diverse diet rich in fresh foods, with lower calories from starchy staples than is normally consumed in LMICs, and higher consumption of nutrient-dense foods, including 575 calories of legumes, nuts, or soy foods.

### 2.3 Developing a “standard approach” baseline: Re-estimating EAT-Lancet reference diet affordability for 2017

Hirvonen et al. (2020) used 2011 ICP price data and purchasing power (PPP) conversion factors for 2011 to estimate CoAHD based on the ELD. They then compared these cost estimates to survey-based estimates of countries' mean daily per capita household income/expenditures from the (now defunct) World Bank's PovcalNet system. Hirvonen et al. (2020) first categorized the 744 food items available in the 2011 ICP into the 15 food groups derived from the 21 ELD food groups, and then used the National Nutrient Database of the United States Department of Agriculture (USDA, 2013) to obtain each item's edible portion and energy content (kcal). After this, they computed the item's price in edible calorie terms and selected the cheapest item in each ELD food group, the aggregate of which is the least-cost ELD expressed in PPP dollars.^7^ Their affordability estimates were computed for 141 countries, accounting for 92 percent of the world’s population.

Here we update these ELD CoAHD estimates by first applying Hirvonen et al.’s (2020) method to 2017 ICP price data and 2017 PPP conversion factors. We use the user-written ‘*pip’* command in Stata (Castaneda, 2023) to compare our CoAHD estimates to countries’ mean daily per capita incomes/expenditures obtained from World Bank’s (2024) Poverty and Inequality Platform (PIP) database.^8^ After restricting the data to countries with reliable PPP conversion factors and income/expenditure distribution data from the World Bank (2024), we are left with 137 countries. Together, these countries represented 91.3 percent of the world’s population in 2017. Tables A2 and A3 in Appendix A list the ICP countries included in and excluded from the main analyses, respectively.^9^

The 2017 ICP has 665 food items that we can use in the estimation. A notable change in the 2017 ICP from its 2011 version is the limited representation of items within the Dark Green Vegetable and Palm Oil food groups. Out of 173 countries, only 35 have a dark green vegetable and 45 have palm oil on their price item list. This necessitated merging these two food groups with Other Vegetables and Oils and Fats, respectively. We also merged Lard/Tallow with Oils and Fats due to its limited availability in the ICP, already in the 2011 ICP series.^10^ This revised food grouping is reported in the last column of Table A1 in Appendix A.

### 2.4 Innovation 1: Developing a Demographic Scaling Factor

As energy requirements vary by age and sex, one limitation of previous CoAHD estimates is that the recommended dietary intakes are set for a 30-year non-pregnant and non-lactating woman as a “representative” consumer. Clearly, this is computationally convenient, but it fails to account for the considerable demographic differences across countries that affect average energy requirements at the country level (see Bai et al., 2020b, for example). Lower income countries are generally characterized by younger populations than richer countries; even the world’s two most populous countries, China and India, have vastly different age-sex structures. As Fig. B1 in Appendix B demonstrates, China’s population is relatively old for its level of development due to stringent family planning laws, while India still has a very young population – indeed, 120 million Indians are under the age of 5, with food intake requirements that will inevitably be much lower than those of a 30-year-old woman. Additionally, energy needs increase during pregnancy and lactation, requiring further adjustments for countries with high-birth rates vis-à-vis countries with low-birth rates.

To explore sensitivity to cross-country differences to age-sex structures, we used the age and sex disaggregated population data for 2015 provided by the United Nation’s Population Division (United Nations, 2019) together with FAO, WHO and UNU (2004) estimates of human energy requirements by age groups to construct a ‘age-sex scaling factor’ that adjusts the kcal per person target proportionally across the ELD groups. First, we calculated the average human energy requirements for seven sex-specific age categories using estimates provided by FAO et al. (2004); see Table B1 in Appendix B. Second, using the same age-sex brackets and data from the United Nation’s Population Division (United Nations, 2019), we estimated the total population in each country by gender and age bracket. Third, we multiplied the number of people in each age-sex bracket with the corresponding human energy requirement estimate and totaled these numbers to calculate the total energy requirement in each country. Finally, we calculated the average energy requirement in each country by dividing the total energy requirement with each country’s total population. Fifth, the age-sex scaling factor was computed by dividing the average energy requirement by 2,500 kcal.

This age-sex scaling factor provides the country’s average energy requirement relative to the average energy needs of a 30-year-old woman weighing 60 kg. The mean age-sex scaling factor is 0.93 among the 137 countries in our dataset, indicating that the average global energy requirement is about 7 percent below of those estimated for a 30-year female. However, as Table B2 and panel A of Fig B2 in Appendix B show, there is a broad spread of scaling factors, including relatively low age-sex scaling factors for some relatively poor and populous countries, such as India (0.94), Pakistan (0.91), Bangladesh (0.94), Nigeria (0.88) and Ethiopia (0.89).

Next, we explore whether an additional adjustment for the higher calorie requirements of pregnant and lactating women matters. To estimate the number of pregnancies in each country in 2017, we used estimates of the crude birth rate (the number of live births per 1000 people) provided by the UN Population Division and applied the following formula Number of Pregnancies = (Crude Birth Rate / 1000) * Population * 9/12, where the last part (9/12) adjusts for the fact that pregnancies typically last for 9 months. As for lactation, we assumed a 12-month breastfeeding period: Number of lactating women = (Crude Birth Rate in the previous year/ 1000) * Population. The available estimates suggest that energy needs during pregnancy increase by 264 kcal (average across the three semesters) (IOM, 2006), implying an 11-percent increase compared to the estimated energy requirements reported in Table B1 in Appendix B. Breastfeeding during the first 12 months increases energy needs by 15 percent (average across 0–6 and 6–12 months postpartum) (IOM, 2006). Using the formulas above we estimated the share of population that was pregnant in 2017 and the share that was lactating in 2017. We then multiplied these shares with the percent increase in energy needs during pregnancy and lactation. We then applied this ‘pregnancy-lactation’ scaling factor to the cost estimates.

Adjustments for the increased needs during pregnancy and lactation matter less than the differences in age-sex profiles. Take Niger, a country with the highest crude birth rate in the world. We estimate that 3.5 percent of Niger’s total population was pregnant in 2017 and 4.7 percent were lactating. Therefore, our cost estimate for Niger is downward biased by 1.1 percent (=11 % * 0.035 + 15 %*0.047). In the average country in our data, 1.5 percent of the total population was pregnant and 2.1 percent lactating in 2017, implying a downward bias of only 0.5 percent. Panel B of Fig. B2 shows the full distribution of the ‘pregnancy-lactation scaling factor’.

### 2.5 Innovation 2: Estimating country-specific non-food basic needs expenditures and household food budgets

While Hirvonen et al. (2020) estimated the number of people who could not afford the ELD if they spent all of their income or expenditure on food, the CoAHD estimates in the annual SOFI reports go a step further by accounting for non-food expenditure requirements, essentially adding a non-food poverty line to a healthy diet food poverty line. For example, the main estimates in the 2020 *State of Food Security and Nutrition in the World* (FAO et al., 2020) assumed that 63 percent of a household’s total income/expenditure could be spent on food, thus assuming a 37 percent allowance for necessary non-food expenditures (e.g., clothing, fuel, housing). Therefore, the total “healthy diet poverty line” in the 2020 SOFI report for any given country is equal to that country’s least-cost healthy diet estimate multiplied by 1/0.63; the higher the healthy diet cost, the higher the non-food poverty line. The most recent 2022 SOFI report instead allows for a 48 percent non-food allowance, or a multiplication factor of 1/0.48 (FAO et al., 2022).

While making some kind of assumption about non-food expenditure requirements is obviously necessary for estimating minimum food budgets in each country, the approaches used in the SOFI reports are problematic for several reasons. A first problem here is that if the healthy diet cost for a particular country is inflated by any kind of measurement error (see below), the non-food “poverty line” is automatically inflated by the same error. Second, this approach imposes average food expenditure shares to remain constant across countries, even though the relative costs between food and non-food basic needs is likely to vary across country income levels (e.g., available housing is generally much more expensive in high income countries). Moreover, non-food poverty lines (basic needs) should also be context-specific (e.g., heating is essential for survival in very cold countries, but irrelevant in very hot countries).

It is admittedly difficult to assess how non-food expenditure poverty lines could or should vary across countries, and clearly there are normative assertions in assessing what is a basic need or not. However, since assuming fixed food and non-food expenditure shares is clearly unsatisfactory, we suggest an alternative approach building on Allen (2017), that aims to systematically estimate non-food basic needs (poverty lines) across countries using ICP non-food price data. Then, in a second stage, we derive a distribution of estimated household food budgets that are simply the residual from deducting Allen-based food expenditure needs from total income/expenditure distributions reported by the World Bank.

Specifically, Allen (2017) devises a budget for non-food basic needs that is the sum of the monetary costs a series of minimal necessities (as Allen defines them) for: (1) housing costs; (2) fuel costs for heating and cooking; (3) lighting costs; (4) clothing costs; and (5) soap costs. Allen sets the minimum requirement for housing at three square meters per person. To estimate the daily costs associated with this requirement, we primarily use rental expense data from the ICP-2017. However, these data for OECD countries are not available in the ICP-2017 and so we resort to rental estimates from major cities, an approach that probably overestimates rental costs in richer countries compared to LMICs. The needs for fuel and lighting vary with the climate. In warmer regions, Allen identifies the minimum lighting as 0.4 million BTUs, and fuel requirement as 1.6 million British Thermal Units (BTUs) aligning with the energy poverty threshold previously established by the Millennium Development Goals. These requirements increase in colder climates. By applying population-weighted heating-degree day (HDDs) estimates from Atalla, Gualdi and Lanza (2018) and Allen's formula that scales fuel and lighting requirements across countries based on HDDs, we estimated the minimum BTU needs in each country and then computed the least-cost of achieving these needs from the energy sources available in the ICP. Clothing requirements also vary with climate, and we used the same approach to estimate the cost of minimum clothing requirements in each country (Allen sets a minimum of 19 m of cloth per person annually in warmer climates). Additionally, Allen calculates the annual soap needs at 25 g per week per person, for which we estimated the daily costs using cost of non-liquid soap available in the ICP data.

Appendix C provides more details about the method we applied and the data sources we used. While Allen’s study was limited to data from just 13 countries, our approach is based on much more extensive 2017 ICP price data, permitting us to estimate these costs for 137 countries.

### 2.6 Sensitivity check 1: Exploring possible problems with low product coverage and urban bias in the ICP food price surveys

The least-cost method selects the cheapest food item as the representative food item within the food group. Consequently, shorter product lists in the price surveys are likely to be associated with higher costs, particularly if the cheapest available energy sources within a food group are not included in the price survey. We explore this issue by analyzing the food group specific product coverage across countries.

We begin by quantifying the potential product coverage bias in each ELD food group by defining the maximum number of foods in that category from the universe of all food items listed in the 2017 ICP. This varies from a high of 57 different starchy staples priced at least once among the 173 countries to just 7 types of sugars/sweeteners and 9 types of oils and fats (Table D3 in Appendix D). For each country-food group combination we then measure the share of food items priced in that country relative to the total number of food items in the 2017 ICP food list. For example, the ICP’s legumes/nuts list includes just 12 items globally, so if a country priced 3 legumes/nuts then its share is 3/12 or 25 percent.

Next, we explore the relationship between product coverage and least-cost estimates, separately for each food group. We do this in two stages. First, since the coverage-cost relationship is likely to be non-linear with costs increasing only once the product coverage share becomes sufficiently low, we use local polynomial regression methods to regress food group specific ELD costs on its product coverage shares. We then use non-parametric regression lines to identify turning points: points below which the product coverage share starts to affect the least-cost estimate for each food group. Second, we apply these visually identified turning points as “knots” in spline regressions in order to estimate regression coefficients that reflect the marginal increase in ELD food group costs once coverage falls below these visually pre-identified thresholds. These linear regressions control for various potentially “true” determinants of ELD costs such as GDP per capita, average rainfall, average temperature, and the quality of transport and infrastructure logistics. Moreover, these regressions also control for potential urban bias measured as the difference between the rural population share in a country in 2017 (World Bank, 2019) and the share of rural shops in total shops providing price quotations in each ICP country (from the 2017 ICP *meta*-data). So, we also use these regression estimates to gauge the role of urban bias in ELD cost estimates based on the ICP data.

In the final step, we use the coefficient on the first spline estimated with the spline regression to adjust the ELD cost estimate in countries that have a product coverage share below the identified turning point. However, we do so only for food groups with a statistically significant coefficient on the product coverage share variable, and only up to a limit of the 25th percentile of the ELD cost distribution so as to avoid ending up with excessively small adjusted cost values. We then sum up all the adjusted food group specific costs to calculate the total adjusted ELD costs for each country.

### 2.7 Sensitivity check 2: Varying physical activity levels and their associated calorie requirements

Caloric requirements also depend on activity levels. Occupational structures across countries vary significantly with income levels, which in turn affects physical activity levels. In poorer countries, a sizable portion of the workforce is typically engaged in quite strenuous non-mechanized agriculture and household chores, while in wealthier countries, the adult population often works in office or service sector jobs characterized by lower energy needs. In one recent innovative study, Srinivasan et al. (2020) estimated that an hour of drudgery reduction could reduce energy requirements by 11–22 percent for men and 13–17 percent for women in Ghana and India.

Making precise assumptions about activity levels in each country is challenging and beyond the scope of this paper, because nationally representative data on physical activity by age and gender is not available. However, we can explore this potential sensitivity by changing assumptions about activity levels. The ELD is benchmarked for a 30-year-old woman with a physical activity level ranging from moderate to high (Willett et al., 2019). The National Academy of Medicine in the US provides Physical Activity Level (PAL) coefficients for four different activity levels: ‘Sedentary’, ‘Low active’, ‘Active’, and ‘Very active’ (IOM, 2006). The academy further notes that active physical level is recommended to maintain good health (IOM, 2006), prompting us not to consider the lower activity levels. Instead, we explore a scenario where we assume everyone’s activity level is ‘Very active’. We map the activity level of the representative woman in the EAT-*Lancet* diet as 'Active' and use the PAL for this activity level as the benchmark to rescale the PAL coefficient for ‘Very active’. The rescaled PAL is for ‘Very active’ is 1.142, implying a 14 percent higher energy needs than the ‘representative EAT-*Lancet* woman’. We then multiply our benchmark least-cost estimates by this rescaled PAL to understand the extent to which assumptions about activity levels affect affordability estimates.

## 3 Results

### 3.1 Deriving a new baseline: A replication of the cost and affordability of the EAT-Lancet reference diet using the 2017 ICP round

Column 1 of Table 1 replicates Hirvonen et al.’s (2020) estimates of the cost and affordability of the EAT-*Lancet* reference diet (ELD) based on 2011 ICP data and 2011 population statistics. To ensure comparability with the main analyses in this paper that are based on the 2017 ICP data, we restrict the data to the 137 countries that appear in the 2017 ICP database and have reliable PPP exchange rates and income/expenditure distribution data in the PIP. According to these 2011 estimates, 1.52 billion people could not afford the EAT-*Lancet* refence diet even if they spent their entire income on food. Column 2 adopts the same methods to the 2017 ICP data, and the latest expenditure/income data from the World Bank and 2017 population statistics. Here we estimate that 1.571 billion people (Table 1, column 2) could not afford the EAT-*Lancet* diet in 137 countries even if all their expenditure or income were spent on food. However, this affordability definition does not consider non-food basic needs, such as housing, heating, and clothing.

**Table 1:** Estimates of the number and share of the population in 137 countries that cannot afford the EAT-*Lancet* reference diet (“ELD-poor”) by ICP round, and with and without adjustment for non-food expenditure needs.

		1. 2011 ICP data no adjustment for non-food expenditure needs		2. 2017 ICP data no adjustment for non-food expenditure needs		3. 2017 ICP CoAHD baseline with adjustment for 48 % non-food expenditure needs	
	N	ELD-poor (millions)	Headcount (%)	ELD-poor (millions)	Headcount (%)	ELD-poor (millions)	Headcount (%)
Global:	137	1521.6	23.6	1571.2	22.7	3170.7	45.8
By income level:							
High income	40	9.4	0.9	9.0	0.8	16.9	1.5
Upper middle income	36	240.0	10.3	109.5	4.5	504.1	20.8
Lower middle income	37	976.3	37.9	1095.6	38.7	2156.5	76.2
Low income	24	296.0	63.5	357.1	64.6	493.2	89.2
By geographic region:							
East Asia and Pacific	13	316.4	14.9	141.4	6.4	587.2	26.5
Europe and Central Asia	44	14.6	1.8	8.7	1.1	35.2	4.2
Latin America and Caribbean	18	51.3	10.4	48.0	9.1	133.1	25.3
Middle East and North Africa	10	28.4	13.0	47.0	18.9	132.6	53.2
North America	2	4.0	1.2	4.2	1.2	5.9	1.6
South Asia	7	610.1	38.4	712.5	41.4	1407.9	81.8
Sub-Saharan Africa	43	496.9	57.4	609.3	59.7	868.9	85.2

Note: This table reports the number and share of people that cannot afford the EAT-Lancet diet (ELD) even if they spent their entire income on food. The estimates in column 1 are based on price data from the 2011 International Comparison Program (ICP) database. The estimates in column 2 are based on price data from the 2017 ICP database. The estimates in column 3 adopt the SOFI 2022 (FAO) approach of assuming that all country populations need to spend at least 48 percent of their income/expenditure on non-food needs.

Hence, column 3 uses the same methods but now applies the 48 percent non-food expenditure share assumption adopted in the SOFI 2022 report (FAO, et al., 2022). In these 137 countries, and by this specific definition, 3.171 billion could not afford the ELD – one third of the sample population. We treat the results in column 3 as a baseline, as it includes standard healthy diet costing methods and the now standard SOFI method for adjusting for non-food expenditure needs.

### 3.2 Innovation 1: How sensitive are healthy diet cost estimates to demographic adjustments?

Taking the estimates reported in column 3 in Table 1 as the new baseline (reproduced in column 1 of Table 2), we next apply our demographic and pregnancy/lactation scaling factors. Demographic adjustments result in an estimate of 3.027 billion people being unable to afford the ELD in these 137 countries (Table 2, column 2) instead of 3.171 billion, a difference of 143 million fewer people than the unadjusted baseline estimates. The regions seeing the largest reduction in ELD-poor from demographic adjustments are East Asia and the Pacific (44.6 million) South Asia (41.1 million), followed by sub-Saharan Africa (32.4 million). These results suggest that adjusting for demographic differences across countries avoids overestimating the number of people who cannot afford a healthy diet in younger and poorer economies in particular. Moreover, demographic structures change over time; for example, Africa’s population will get older in coming decades, resulting in higher dietary costs (all else equal).

**Table 2:** Revised estimates of the number and share of the population in 137 countries that cannot afford the EAT-*Lancet* reference diet with and without demographic re-scaling.

		1. CoAHD baseline (no demographic re-scaling)		2. CoAHD with demographic re-scaling		3. Difference (column 2 minus column 1)	
	N	ELD-poor (millions)	Headcount (%)	ELD-poor (millions)	Headcount (%)	ELD-poor (millions)	Headcount (% points)
							
Global:	137	3170.7	45.8	3027.4	43.7	−143.3	−2.1
By income level:							
High income	40	16.9	1.5	15.9	1.4	−1.0	−0.1
Upper middle income	36	504.1	20.8	460.0	19.0	−44.1	−1.8
Lower middle income	37	2156.5	76.2	2073.8	73.3	−82.7	−2.9
Low income	24	493.2	89.2	477.7	86.4	−15.5	−2.8
By geographic region:							
East Asia and Pacific	13	587.2	26.5	542.6	24.5	−44.6	−2.0
Europe and Central Asia	44	35.2	4.2	31.3	3.8	−3.9	−0.4
Latin America and Caribbean	18	133.1	25.3	122.6	23.3	−10.5	−2.0
Middle East and North Africa	10	132.6	53.2	121.6	48.8	−11.0	−4.4
North America	2	5.9	1.6	5.9	1.6	0.0	0.0
South Asia	7	1407.9	81.8	1366.8	79.4	−41.1	−2.4
Sub-Saharan Africa	43	868.9	85.2	836.5	82.0	–32.4	−3.2

*Note*: This table reports the number and share of people that cannot afford the EAT-*Lancet* reference diet (ELD) while factoring in a non-food cost component. All estimates are based on price data from the 2017 International Comparison Program (ICP) database and factor in a country specific ELD costs and fixed 48 percent non-food cost component. Column 1 reproduces estimates reported in column 2 of Table 1. The estimates reported in column 2 build on column 1 but apply a demographic scaling factor to ELD costs expenditures.

### 3.3 Innovation 2: How sensitive is the affordability of healthy diets to more systematic estimation of non-food basic need expenditures?

Table 3 reports ELD food expenditure requirements (the least cost of a healthy diet), followed by non-food expenditure requirements based on our extension to Allen’s method, while the last column reports the non-food budget in the total “poverty line”. The cost of a healthy diet is $2.90 per day in 2017 PPP terms and varies moderately over World Bank income levels and regions. The average non-food expenditure requirements are lower than they are for food, at $2.05 per day. However, unlike food, non-food expenditure requirements are much higher in the richest countries compared to LMICs, and also vary by region, being especially low in sub-Saharan Africa ($0.86). Fig. C1 in Appendix C also looks at the association between food and non-food expenditure requirements. The SOFI approach implicitly assumes the two are linearly related. However, the Allen-extension method shows that non-food costs tend to decrease as ELD costs increase, although there is clearly also substantial variation in this association.^11^

**Table 3:** Cost of EAT-Lancet reference diet, cost of non-food needs as defined by Allen (2017), and the share of the non-food budget in total poverty line.

	N	ELD food expenditure requirements (2017 $PPP)	Non-food expenditure requirements, Allen-based method (2017 $PPP) ^a^	Non-food budget in total poverty line, Allen-based method (%)
Global:	137	$2.90	$2.05	41.4
By income level:				
High income	40	$2.58	$3.55	57.9
Upper middle income	36	$3.08	$1.99	39.3
Lower middle income	37	$3.09	$1.19	27.8
Low income	24	$2.85	$0.98	25.6
By geographic region:				
East Asia and Pacific	13	$3.22	$1.87	36.7
Europe and Central Asia	44	$2.67	$3.76	58.5
Latin America and Caribbean	18	$3.15	$1.37	30.3
Middle East and North Africa	10	$2.92	$1.43	32.9
North America	2	$2.50	$3.82	60.4
South Asia	7	$3.27	$1.09	25.0
Sub-Saharan Africa	43	$2.87	$0.86	23.1

*Note*: a. These are country-specific non-food cost components based on the approach described in Allen (2017).

The last column in Table 3 shows that the average non-food budget in the total “poverty line” is about 41 percent globally, in between the 37 percent share in the 2020 SOFI report and the 48 percent share in the 2022 SOFI report. However, this share increases sharply with a country’s average income levels, from $0.98/day in low-income countries to $3.55/day in high income countries. Hence, this adjustment to healthy diet affordability estimate is likely to matter not only in terms of total global levels of the share of people unable to afford a healthy diet, but it will also change results for specific countries and regions.

In column 1 of Table 4 we reproduce the estimates reported in column 3 in Table 3 as the new baseline that incorporates the demographic scaling factors and the SOFI 2022 approach of incorporating a 48-percent allowance for non-food expenditures. We estimate that the incomes of 3.027 billion people are below this nutritional poverty line, similar to the SOFI 2022 estimates of 3.1 billion using a more generic healthy diet. When we use our extension of the Allen (2017) approach to generate country-specific non-food expenditure allowances the number of people whose incomes fall below the nutritional poverty line is 2.127 billion (column 2 of Table 3), about 899.9 million fewer than the SOFI fixed non-food expenditure approach reported in Column 1 of Table 4.

**Table 4:** Adding country-specific non-food cost components to estimates of the number and share of the population in 137 countries that cannot afford the EAT-*Lancet* reference diet (by country income level and geographic region).

		1. A 48 % fixed non-food cost component		2. A country-specific non-food cost component		3. Difference (column 2 minus column 1)	
	N	ELD-poor (millions)	Headcount (%)	ELD-poor (millions)	Headcount (%)	ELD poor (millions)	Headcount(%-points)
Global:	137	3027.4	43.7	2127.5	30.7	−899.9	−13.0
By income level:							
High income	40	15.9	1.4	20.7	1.9	4.8	0.5
Upper middle income	36	460.0	19.0	294.6	12.1	−165.4	−6.9
Lower middle income	37	2073.8	73.3	1391.5	49.2	−682.3	−24.1
Low income	24	477.7	86.4	420.6	76.0	−57.1	−10.4
By geographic region:							
East Asia and Pacific	13	542.6	24.5	327.8	14.8	−214.8	−9.7
Europe and Central Asia	44	31.3	3.8	48.5	5.9	17.2	2.1
Latin America and Caribbean	18	122.6	23.3	89.8	17.1	–32.8	−6.2
Middle East and North Africa	10	121.6	48.8	72.0	28.9	−49.6	−19.9
North America	2	5.9	1.6	6.8	1.9	0.9	0.3
South Asia	7	1366.8	79.4	882.0	51.2	−484.8	−28.2
Sub-Saharan Africa	43	836.5	82.0	700.6	68.7	−135.9	−13.3

*Note*: This table reports the number and share of people that cannot afford the EAT-Lancet reference diet (ELD) while factoring in a non-food cost component. All estimates are based on price data from the 2017 International Comparison Program (ICP) database. The estimates reported in column 1 incorporate 48-percent allowance to non-food expenditures based on the country specific ELD costs. The estimates reported in column 2 are based on country specific ELD costs and country specific non-food cost component based on an extension of the approach described in Allen (2017).

There are large declines in the number and prevalence of “diet poor” across all regions except the Europe and Central Asia where “diet poverty” actually increases because of the region’s high non-food expenditure needs (high rents, but also higher heating and clothing costs associated with colder climates). In South Asia almost half a billion fewer people are classified as diet poor, while in East Asia and the Pacific there is a reduction of 214.8 million, and a reduction of 135.9 million people in sub-Saharan Africa.

**Fig. 1:**
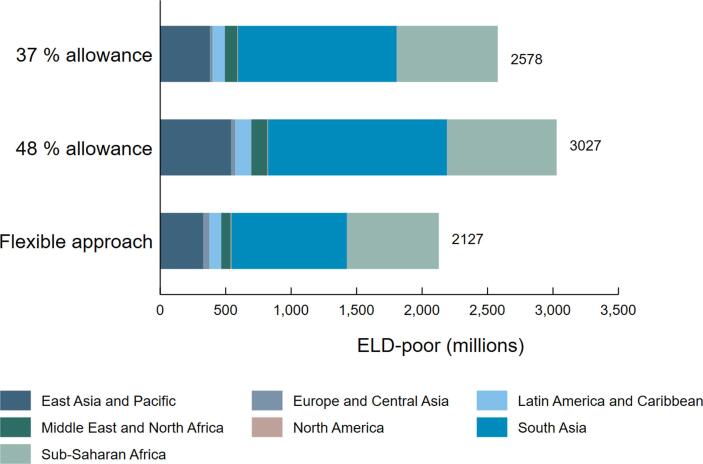
Number of people who cannot afford the EAT-Lancet reference diet (ELD), by country geographic region and by non-food cost allowance method. Note: “37 % allowance” refers to estimates based on 37-percent allowance to non-food expenditures based on the country specific ELD costs as in SOFI-2020 and “49 % allowance” refers to corresponding non-food allowance used in SOFI-2022. “Flexible approach” refers to estimates based on country specific ELD costs and country specific non-food cost component based on an extension of the approach described in Allen (2017).

Fig. 1 further highlights the enormous sensitivity regarding the assumptions made about the non-food expenditures allowances by comparing the number of ‘diet poor’ individuals in each scenario: a 37-percent non-food allowance as in SOFI 2020, a 48-percent non-food allowance as in SOFI 2022, or the flexible approach based on the Allen method. Hence if one broadly accepts the validity of this extension of Allen’s (2017) approach for estimating non-food expenditure requirements, global estimates for the share of people who cannot afford a healthy diet are reduced quite substantially.

### 3.4 Sensitivity check 1: Do limitations in the ICP food price surveys inflate healthy diet costs?

As we noted above, although the ICP has extensive country coverage, some participating countries have relatively short food lists that could inflate least-cost healthy diet estimation if cheap foods are not represented. Fig. D1 in Appendix D reports the frequency distribution of product price quotations (for all foods) in each of the ICP countries in our sample, while Table D1 lists the number of items for each country while Table D2 does so both in aggregate and for each of the ELD food groups. At one extreme, the most populous countries in Asia typically price a large number of foods, including India (2 0 8 items), China (1 9 4 items), Pakistan (1 8 7 items), Bangladesh (1 7 1 items), Vietnam (1 6 1 items) and Indonesia (1 5 5 items). However, over half the ICP country sample report prices for fewer than 100 food items, with product coverage being particularly poor in Latin America and the Caribbean. Some relatively large countries with short product lists are Brazil and Mexico (78 items in both countries), Colombia and Ecuador (67 items), while in Africa only Sudan (75 items) and South Africa (62) stand out as large countries with short food lists. Across food groups, coverage of legumes is in particular need of improvement given their importance to diets in Latin America and sub-Saharan Africa, but coverage is also sometimes lower for fruits, vegetables, and animal-sourced foods.^12^

To check whether poor product coverage is associated with higher estimated ELD costs, Fig. D2 in Appendix D presents local polynomial regression plots of ELD costs for each country and each food group against the share of all possible foods that are priced in a country. The vertical red lines, from left to right, represent the 10th, 25th, 50th, 75th and 90th percentiles, and are shown to demarcate any potential turning points or asymptotes for subsequent use in spline regressions. In most instances – except oils and sugar^13^ – there are signs of a negative gradient between ELD food group costs and corresponding food group coverage, though this negative gradient ceases at various asymptotes, depending on the food group. Overall, this visual evidence suggests that product coverage bias could be a significant concern for several food groups, although the curvatures and gradients of these curves are diverse, as one might expect.

What about urban bias in the food vendors surveyed? Appendix Table D4 reports the share of ICP countries that survey both rural and urban areas, and – if rural vendors are surveyed – the share of rural vendors surveyed among all vendors. Most upper middle- and high-income countries do not survey rural vendors at all, whereas all South Asian countries survey rural areas, although in those that did so only 25 percent of shops surveyed were rural despite around two thirds of the population being rural. In Sub-Saharan Africa just over half of countries surveyed any rural areas, but in those that did so only 16 percent of vendors were located in rural areas. Surprisingly, half of all low-income countries – where the average rural population share is 66 percent – did not sample any rural vendors. Thus, while the ICP has engaged in re-weighting to correct for urban bias in many instances, that was presumably difficult for a large number of LMICs that had no rural price information to adjust. This suggests that some of the prices in the ICP could indeed be “urban biased” and Appendix Fig. D3 provides some suggestive evidence that urban biased food surveys are associated with higher food group specific costs, though only in specific instances.

These bivariate results on productive coverage bias and urban bias, but Appendix Table D5 reports robust multivariate regressions^14^ that include a spline regression function for product coverage bias, the extent of urban bias in survey coverage, and the control variables described above. The results in Table D5 show that shorter product lists – up to an asymptote – are indeed associated with higher ELD costs for starchy staples, legumes/nuts, vegetables, dairy and poultry products and fish; whereas oils, sugars, fruits, and red meat have no indications of significant slope coefficients, while the sugars food group has an unexpected but modest positive slope coefficient.

To interpret the magnitudes of the coefficients, predictions from these coefficients are presented below the main regression results in Table D5 for the hypothetical example of moving from the minimum level of food coverage gap to the asymptote specific for each food group. Moving from the minimum coverage to the turning point reduces the least-cost starchy staple estimates by $0.18, with analogous reductions of $0.20 for legumes/nuts, $0.46 for vegetables, $0.19 for dairy and $0.18 for poultry and fish products. However, since the asymptotes for starchy staples and vegetables are the 25th and 10th percentiles, respectively, this “bias” only affects a relatively small subset of countries for these food groups. In contrast, the asymptote for legumes/nuts is the 75th percentile, so adjustments for poor coverage of this food group affect most countries. Gaps in coverage are correlated across ELD food groups (Appendix D Table D6), implying that the predicted impacts of short food lists at the country level will be partially additive in many countries, such that the total upward adjustment of the cost of the ELD diet could be sizable in those countries with short overall food lists.

The coefficients on the gap in the urban coverage measure in Table D5 are only significant and positive coefficients for dairy and fruit, but even these two coefficients are not robust to excluding high income countries from the sample (none of whom survey rural areas). Hence the size of any urban bias in LMICs seems a minor issue from a least-cost diet perspective. In summary, the regression results from Table D5 suggest that short product lists likely bias upwards the cost of several ELD food groups, whereas there is no robust evidence that urban biases affect diet cost estimates.

To assess the potential influence of product coverage bias on CoAHD estimates, we use the product coverage coefficients from Table D5 to adjust costs for any food group with a statistically significant coefficient, and then use those adjusted food group costs to aggregate up to an adjusted total ELD costs that is then used to re-calculate ELD affordability estimates (see the description in Appendix D). Across all 137 countries, the total ELD cost only falls marginally, from $2.86 to $2.72 in 2017 PPP dollar terms, with half of this stemming from imposed reductions in the legumes/nuts food group costs for which product coverage is often quite poor (Appendix Table D6). In specific regions, however, the cost reductions are much larger, especially Latin America (29 cents). While these adjustments sometimes appear modest, it is worth noting that 29 cents per day for a family of five – the Latin American example – amounts to around 40 PPP dollars per month, which is sizable.

Column 1 of Table 5 first reports the affordability estimates that incorporate the country specific non-food cost component based on Allen (2017) and the demographic scaling factor adjusted estimates reported in Column 2 of Table 4, while Column 2 makes adjustments for low product coverage using the approach described in Appendix D. Adjustments for low product coverage results in an estimated 2.065 billion people being unable to afford a healthy, a decline of 62.8 million from the new benchmark (Column 3 of Table 5). The country- and region-level changes in absolute numbers of diet-based poor in Column (3) are a function of low product coverage (i.e. larger diet cost adjustments) as well as the prevailing distribution of income/expenditure in a country (i.e., how many people are near the diet-based poverty line) and the population of the country. Hence the largest reductions in diet-based poor in absolute terms are in Latin America and the Caribbean (20 million) which has poor product coverage but higher incomes/expenditures, and sub-Saharan Africa (19.3 million) where product coverage gaps are modest, but incomes/expenditure are low.

**Table 5:** Revised estimates of the number and share of the population in 137 countries that are poor according to an EAT-*Lancet* reference diet food poverty line and country-specific non-food poverty lines, without and with adjustments for potential urban and product coverage biases.

		1. New benchmark (column 2 of Table 4)		2. Estimates that adjust for low product coverage		3. Difference (column 2 minus column 1)	
	N	ELD poor (millions)	Headcount (%)	ELD poor (millions)	Headcount (%)	ELD poor (millions)	Headcount (%-points)
Global:	137	2127.5	30.7	2064.7	29.8	−62.8	−0.9
By income level:							
High income	40	20.7	1.9	18.2	1.6	−2.6	−0.2
Upper middle income	36	294.6	12.1	265.1	10.9	−29.5	−1.2
Lower middle income	37	1391.5	49.2	1369.1	48.4	–22.4	−0.8
Low income	24	420.6	76.0	412.4	74.6	−8.2	−1.5
By geographic region:							
East Asia and Pacific	13	327.8	14.8	324.2	14.6	−3.6	−0.2
Europe, US & Central Asia	44	48.5	5.9	41.0	5.0	−7.5	−0.9
Latin America and Caribbean	18	89.8	17.1	69.3	13.2	−20.4	−3.9
Middle East and North Africa	10	72.0	28.9	64.9	26.1	−7.1	−2.9
North America	2	6.8	1.9	6.0	1.6	−0.8	−0.2
South Asia	7	882.0	51.2	877.9	51.0	−4.0	−0.2
Sub-Saharan Africa	43	700.6	68.7	681.4	66.8	−19.3	−1.9

*Note*: This table reports the number and share of people that cannot afford the EAT-*Lancet* reference diet (ELD) while factoring in a non-food cost component. All estimates are based on price data from the 2017 International Comparison Program (ICP) database and factor in a country specific ELD costs and country specific non-food cost component based on an extension of the approach described in Allen (2017). Column 1 reproduces estimates reported in column 2 of Table 4. The estimates reported in column 2 build on column 1 but adjust ELD costs for potential urban and product coverage biases in 2017 ICP data. See Appendix D for a description of how estimates are adjusted for low product coverage.

### 3.5 Sensitivity check 2: Varying physical activity levels and calorie requirements

Table 6 takes the estimates from column 2 of Table 4 (shown as column 1 in Table 6) and contrasts these with rescaled diet cost estimates under the assumption that everyone adheres to a 'Very active' lifestyle (presented in column 2 of Table 6). By assuming a very active lifestyle, which implies higher energy needs for all, the estimated global diet poverty headcount increases to 2.5 billion people, or 36 percent. This represents an increase of 451 million people or an additional 6.5 percentage points compared to the benchmark estimates reported in column 1.^15^

**Table 6:** Revised estimates of the number and share of the population in 137 countries that are poor according to an EAT-*Lancet* reference diet food poverty line and country-specific non-food poverty lines assuming very high activity levels by country income level and geographic region.

		1. New benchmark (column 2 of Table 4)		2. Assuming very high activity level for all		3. Difference (2.-1.)	
	N	ELD poor (millions)	Headcount (%)	ELD poor (millions)	Headcount (%)	ELD poor (millions)	Headcount (%-points)
Global:	137	2127.5	30.7	2578.4	37.3	450.9	6.5
By income level:							
High income	40	20.7	1.9	23.8	2.1	3.1	0.3
Upper middle income	36	294.6	12.1	388.4	16.0	93.8	3.9
Lower middle income	37	1391.5	49.2	1706.7	60.3	315.1	11.1
Low income	24	420.6	76.0	459.5	83.1	38.9	7.0
By geographic region:							
East Asia and Pacific	13	327.8	14.8	428.2	19.3	100.4	4.5
Europe, US & Central Asia	44	48.5	5.9	59.0	7.1	10.4	1.3
Latin America and Caribbean	18	89.8	17.1	109.5	20.8	19.8	3.8
Middle East and North Africa	10	72.0	28.9	96.3	38.7	24.3	9.7
North America	2	6.8	1.9	7.6	2.1	0.8	0.2
South Asia	7	882.0	51.2	1095.9	63.7	213.9	12.4
Sub-Saharan Africa	43	700.6	68.7	781.9	76.7	81.3	8.0

*Note*: This table reports the number and share of people that cannot afford the EAT-*Lancet* reference diet (ELD) while factoring in a non-food cost component as well as adjustments. All estimates are based on price data from the 2017 International Comparison Program (ICP) database and factor in a country specific ELD costs and country specific non-food cost component based on an extension of the approach described in Allen (2017). They also adjust for differences in demographic profiles across countries and potential urban and product coverage biases in 2017 ICP data. Column 2 considers a scenario where all people follow a ‘very active’ lifestyle requiring minimal energy needs and up-weighs the energy requirements in the ELD accordingly.

Could – and should – future analyses adjust for differing calorie requirements across populations? Arguably so. While there is a paucity of data on physical activity levels and time use (Srinivasan et al., 2020), the fact that physical activity levels are generally higher in poorer than in richer countries means that a constant energy requirement assumption for all countries is not conceptually appealing.

## 4 Conclusions

Previous studies have documented a fact long suspected, but until recently never quantified: that vast numbers of people in LMICs world cannot afford a healthy diet. In this study we explored several important innovations and extensions for improving CoAHD metrics at the global level, although many of these could also be applied to regional, national, or even subnational studies.

Our first key finding is that demographic differences across countries have substantial impacts on CoAHD estimates because many populous and relatively poor countries also have young populations (e.g., India, Nigeria), resulting in a sizable decrease in our “global” estimates of the number of people who cannot afford a healthy diet. This demographic scaling factor is accessible in Appendix D and easy to apply.

Our second key finding is that analysts need to apply much more care in deriving actual food budgets in global analyses; using fixed non-food expenditure requirements to do so is clearly unsatisfactory and distorts inter-country comparisons. Extending and improving upon Allen’s (2017) using ICP *non*-food price data results have a major influence on global estimates and cross-country comparisons. Of course, this Allen-extension approach is not immune to criticism (see Ferreira, 2017, Ravallion, 2017) but at least it has an economic logic and provides a tractable method for estimation and refinement. In any case, we show that there is tremendous sensitivity of healthy diet affordability estimates to the assumptions and methods used to estimate *non-*food basic needs. This warrants much closer attention in global estimates.

Our third key finding is that low product coverage may inflate healthy diet cost estimates in those countries – often in Latin America – with short food item lists. In particular, coverage of legumes/nuts products in the ICP is quite limited, which is problematic since this food group features prominent in healthy diet recommendations (Willett et al., 2019) and also in the actual diets of poor populations in LMICs. Our results on urban bias in price surveys seem less concerning, but the representativeness of ICP surveys is a subject warranting further research, especially in a CoAHD context.

### 4.1 Limitations of this study’s data and analytical methods

Despite these extensions to existing methods, there are limitations to this study in terms of data quality and the range of analyses we were able to conduct.

First, our study uses the ELD, which is not without criticism, some of it scientific in nature, much of it not. On the scientific front, Beal et al. (2023) find that ELD diets could result in inadequate micronutrient intake. Others criticize the ELD’s stringent restrictions on nutrient-dense animal sourced foods for undernourished LMIC populations that also culturally prioritize animal sourced foods (Adesogan et al., 2020). However, it is worth pointing out that if LMIC populations shifted to an imperfect EAT-*Lancet* reference diet, that shift would result in dramatic improvements in nutrient intake and overall health for the average LMIC consumer because current diets are grossly imbalanced (Headey et al., 2023, Pauw et al., 2023).

Third, our study (and all global CoAHD studies) uses price and income/expenditure data that are unfortunately quite out of date and also highly aggregated. A new 2021 ICP round will shortly provide updated food and non-food price data and there are proposals to make ICP price surveys data annual. Regular ICP price updates would be ideal because the current SOFI approach of inflating past prices (e.g., 2017) forward using general consumer food price indices is far from ideal. Equally problematic is the dearth of recent household income and expenditure surveys in many LMICs, especially India, where the last national survey was conducted in 2011–12 (we note, however, that a new survey was completed in 2022, although the data are yet to be released). There is also an urgent need for country-level CoAHD analyses using richer time series price, income, or wage data (Headey et al., 2023), as well as household expenditure surveys (e.g., Raghunathan et al., 2021). Those studies offer scope for disaggregation by subnational regions, by rural/urban locations, gender, occupations, and other socioeconomic strata, as well as scope for analyses of trends and seasonality fluctuations and various simulations and policy analyses (e.g., social protection).

### 4.2 Possible extensions to CoAHD methods

This study analyzed several important and tractable limitations of existing CoAHD methods, but there are other possible extensions that could be pursued in future work, albeit with more daunting technical challenges.

First, least-cost healthy diets are hypothetical and run the risk of being unacceptable to actual poor consumers. Definitions of food security (FAO, 1996, Barrett, 2010) and monetary poverty (Ravallion, 2000) both emphasize acceptability or consistency with preferences. To explore how food preference constraints affect healthy diet costs, Mahrt and colleagues use household expenditure survey data from Myanmar to weigh food items within each food group by each item’s food group-specific consumption share to produce preference-adjusted healthy diet costs (Mahrt et al., 2022; Mahrt et al., 2019). In Myanmar, one striking finding was strong preferences for animal source foods over pulses in the “proteins” food group. More generally, incorporation of preferences would obviously increase the monetary value of “least-cost” heathy diets. However, whether one should incorporate food preferences in healthy diet costs is ultimately a normative question (Mahrt et al., 2022).

Second, meal preparation costs may also add to the real daily cost of a diet and be a latent component of food preferences (Masters et al., 2021), but measuring meal preparation costs is challenging. Although the ICP reports fuel costs to some extent, these would vary within countries across different types of households and vary across different types of meals. In addition, one would need realistic estimates of opportunity costs for meal preparation (e.g., foregone wages/income multiplied by time spent on cooking). Meal preparation responsibilities typically fall on women, but these opportunity costs would also vary across households and require time use data and appropriate wage/income data.^16^

Third, economists have quantified economies of scale in household expenditure (Deaton and Paxson, 1998, Lanjouw and Ravallion, 1995). With food this could stem from savings associated bulk purchases at lower unit costs, as well as unmeasured food preparation costs. For non-food expenditures there could be considerable savings on housing and utility costs, among others. In theory, one would expect adjustments for scale economies to reduce estimates of the number of diet poor people at the global level, because poorer countries – and poor populations within those countries – have larger family sizes than high income countries. While this could be an important area for future research, it is not straightforward because the extent of savings from household size economies could be highly context specific. One study from long-run historical data in the US found that scale economies by expenditure groups changed in unanticipated ways over time (Logan, 2011), suggesting potential for a high degree of context-specificity. Another recent study found scale economies in Mexico but not Bangladesh (Calvi et al., 2023).

Fourth, food losses in value chains are presumably factored into the ICP retail prices already, but households themselves can waste food. Our estimates do not factor in food waste within households, which may add to the true costs of a healthy diet, especially as many healthy foods are perishable. A major challenge in this context is documenting how food waste varies across socioeconomic strata. On the one hand, it might be expected that poorer households waste less food, especially relatively expensive foods, but on the other hand they also often lack access to refrigeration (although they may alter their shopping behaviors accordingly). Whether one ought to factor in food waste is itself debatable in a CoAHD context, but there is also a significant empirical challenge. A *meta*-analysis of food waste studies by the UN revealed that the majority of studies come from high-income countries (UNEP, 2021). Indeed, only 2 of 52 studies in their review were sourced from low-income countries.

Fifth, the demographic adjustment factor we develop based on age- and gender-specific calorie requirements is a significant improvement over unadjusted method. However, it could potentially be improved to adjust for differences in weight and height distributions across countries, as well as physical activity levels.

Sixth, while our scaling of diet costs proportional to calorie requirements at different ages or for pregnancy indicates the general sensitivity and direction of these adjustments, the scaling has a tacit assumption that all nutrient requirements are adjusted in the same proportion as energy requirements. This is imprecise, although instructive. Future research can explore costs of reference diets by age using more granulated diets.

Seventh, researchers could explore the fact that poor people shop in different kinds of outlets and may face systematically different prices, as well as different product quality. In theory it may be possible for the ICP and other institutions to construct price estimates more representative of poorer consumers.

### 4.3 Implications for policy and policy analysis

Whether the “true” number of people who cannot afford a healthy diet is 2 billion or 3 billion, or some other number, we can be highly confident that all such estimates are much larger than the 659 million people the World Bank estimates to be extremely poor, or the 702–828 million the FAO deemed to be calorie deprived (hungry) in 2021; but much closer to the several billion people (imprecisely) estimated to have at least one micronutrient deficiency. For example, Stevens et al. (2022) estimate the global prevalence of deficiency in at least one of three micronutrients to be 56 percent among pre-school aged children and 69 percent among non-pregnant women of reproductive age. Those results suggest that several billion people worldwide have at least one micronutrient deficiency. While micronutrient deficiencies can certainly exist because people choose not to consume healthy foods, CoAHD analyses suggest the inability to afford a healthy diet is one likely explanation for vast numbers of people. The scale of the healthy diet affordability challenge is therefore immense, and policymakers need to acknowledge and respond to the scale of this problem.

However, estimation methods *do* matter in terms of their scope to significantly alter global, regional, and national level estimates of the numbers and prevalence of people unable to afford a healthy diet, as well as attempts to estimate the steps required to redress this affordability problem (e.g., social protection costs, economic growth targets, price subsidies, agricultural investments, et cetera). CoAHD metrics have already been used in global simulation models to assess the impacts of COVID-19 (Laborde et al., 2021) and various strategies for repurposing agricultural policies for nutrition (FAO et al., 2022). Some LMIC governments have also started tracking CoAHD metrics, including two of Africa’s largest countries, Ethiopia (Alemayehu et al., 2023) and Nigeria (NBS, GAIN, 2023). This national adoption of CoAHD metrics is encouraging, but we urge governments, international agencies, and analysts to facilitate more timely measurement and dissemination of these metrics through greater investment in more frequent price, wage, and income/expenditure surveys. More data can facilitate more timely and accurate measurement of the healthy diet affordability problem, and more peer-reviewed research on CoAHD methods. More rigorous analyses of those data can then improve policy analysis and provide a stronger scientific basis for nutrition-sensitive development strategies, as well as more accurate targeting of resources.

### CRediT authorship contribution statement

**Derek Headey:** Writing – review & editing, Writing – original draft, Visualization, Validation, Resources, Project administration, Methodology, Investigation, Funding acquisition, Formal analysis, Data curation, Conceptualization. **Kalle Hirvonen:** Writing – review & editing, Writing – original draft, Visualization, Validation, Methodology, Investigation, Formal analysis, Data curation, Conceptualization. **Harold Alderman:** Writing – review & editing, Writing – original draft, Methodology, Investigation, Conceptualization.

## Declaration of Competing Interest

The authors declare that they have no known competing financial interests or personal relationships that could have appeared to influence the work reported in this paper.

## Footnotes

1 Headey and Alderman (2019) use ICP *meta*-data to see whether urban biases affect the relative caloric prices of different food groups but find little evidence of bias. Forthcoming work from the Food Prices for Nutrition project compares healthy diet costs from ICP data to costs estimated from national sources (personal communication).

2 At this stage in the ICP data collection process the exact steps undertaken in each country are somewhat unclear, and it appears that some agencies likely match prices of food items that they are already collecting through their regular consumer price surveys to those in the regional ICP food lists, while other national agencies might make special efforts to include additional food items for the ICP’s purposes.

3 According to the ICP (World Bank, 2013, p. 27), “Two or more products are said to be comparable either if their physical and economic characteristics are identical, or if they are sufficiently similar that consumers are indifferent between them”.

4 In Ethiopia, for example, the CPI is constructed from a survey of 120 markets, but the government defines all these markets as urban localities, whereas most rural Ethiopians would buy foods from much smaller local markets.

5 In addition to some of the procedures described above, the ICP conducts several data quality assurance processes at the national level, the regional (inter-country) level and at the global (inter-regional) level.

6 There are some controversies regarding the suitability of the EAT-*Lancet* diets for LMICs, because the reference diet was not designed for young children in LMICs who are often facing multiple micronutrient deficiencies and problems with protein quality, for example. The original report by Willett et al. (2019) acknowledges this to some extent, however a recent study by Beal et al., 2023 shows that the EAT-*Lancet* diets result in inadequate micronutrient intake for several key nutrients such as iron, zinc, calcium and B12, and recommended higher reference intakes of animal sourced foods and lower intakes of foods high in phytates.

7 In rare cases where a country reported no prices for any item within the food group, we imputed their cost as the mean price from a country’s geographical sub-region.

8 We omitted income/expenditure surveys conducted during the COVID-19 pandemic (i.e., after 2019) and then selected, for each country, the income/expenditure survey year closest to 2017.

9 The list of ICP countries excluded from the analyses (see Table A3) is dominated by high income and upper-middle income countries where the risk of ELD poverty is likely to be relatively low. There are 5 low-income countries and 10 lower middle-income countries. The total population in these 15 countries is 204 million people. Assuming all people in these countries are ELD poor would increase our estimates by 204 million, representing a 9.6% increase in the global diet poverty headcount compared to the estimate in Table 4, column 2.

10 In Table A4 of Appendix A, we use the 2011 ICP data to apply the same adjustments to check the impact on the ELD cost estimates. Reducing the number of food groups from 15 to 11 decreases the global ELD estimate by $0.23 (measured in 2011 PPPs) – or 8.1 percent, with the largest reductions observed in High-Income countries and the East-Asia and Pacific region (both $0.36).

11 We can be confident in this assertion because the least cost ELD and the least cost healthy diet used in the SOFI reports will be highly correlated with each other, and because costs of healthy diets in the SOFI report do not rise steeply with average incomes.

12 Surveying a sufficiently diverse set of foods is a global challenge for the ICP, but particularly problematic in Latin America, especially in the case of legumes, which are an important part of the diet in this region. Mexico prices four foods in this category; Brazil, Colombia, Ecuador, and Honduras just three, while Paraguay, Nicaragua, Bolivia, and Costa Rica price just two, and El Salvador did not price any food in this category. Among fruits, vegetables, and animal sourced foods the coverage looks better but the “universe” of these foods is very large, as indeed it is with fish. Sub-Saharan Africa likely also has rather low coverage of legumes/nuts, but there are also problems with the legume/nut products selected for pricing in ICP surveys in Africa because they often excluded important local varieties. For example, Ethiopia's ICP list includes groundnuts/peanuts, peas, “spotted beans” and tinned white beans (which are largely irrelevant for rural consumers), but in reality, Ethiopians consume a much wider arrange of legumes, including fava beans, chickpeas, lentils, haricot beans, pigeon pea and mung beans (Atnaf, Tesfaye and Dagne, 2015). Coverage of vegetable products is also potentially low in sub-Saharan Africa given the huge diversity of vegetables consumed across the continent.

13 Given that we would not expect product coverage shares to affect costs for oils or sugars, non-negative gradients for these two food groups provide a placebo test of sorts. These two products are also highly traded, although sugar prices are also subject to high rates of subsidies (Anderson, 2009).

14 Robust regression down-weighs influential outliers or extreme values. We used the *rreg* command in Stata to estimate these robust regressions.

15 For completeness, Table E1 in Appendix E reports the corresponding estimates based on a scenario where all people follow a ‘sedentary’ lifestyle requiring minimal energy needs and down-weighs the energy requirements in the ELD accordingly. By assuming a sedentary lifestyle, the estimated global diet poverty headcount decreases to 1.75 billion people, or 25 percent. This represents a decrease of 376 million people or 5.4 percentage points compared to the benchmark estimate. However, such sedentary lifestyles are not recommended (IOM, 2006) and hence we do not report these estimates here in the main text.

16 However, while country case studies on meal preparation costs might be feasible, it is hard to envisage there being sufficient data to make satisfactory estimates at a global level.
