# Supplementary material for: Estimating the cost and affordability of healthy diets: How much do methods matter?
Source: Food Policy. 2024 Jul;126:102654. doi: 10.1016/j.foodpol.2024.102654 (PMC11287492; doi:10.1016/j.foodpol.2024.102654)
Supplement: Supplementary Data 1 [file mmc1.pdf]

Appendices

Contents

Appendix A. Additional details about the EAT-Lancet diet and International Comparison Program (ICP) data 3

Table A1. Composition of the EAT-*Lancet* reference diet (ELD), by food group 4

Table A2. The 137 ICP countries included in the main analyses 5

Table A3. The ICP countries not included in the main analyses 9

Table A4. Least ELD cost based on ICP-2011 in 137 countries, based on 15 food groups versus 11 food groups........ 11

Appendix B. Additional details on the demographic scaling factor 12

Figure B1. Differences in the population “pyramids” between China and India........ 13

Table B1. Estimated human energy requirements 14

Figure B2. Distributions of the demographic scaling factors across the 137 countries in our data 15

Table B2. Estimated demographic scaling factor, by country 16

Appendix C. Additional details about non-food budgets 20

Housing cost needs 20

Fuel cost needs for heating & cooking, and lighting 20

Clothing cost needs........ 21

Soap cost needs........ 21

Figure C1. Association between cost of the food and non-food budgets........ 22

Appendix D. Correcting for urban bias and short product lists in the ICP data, additional exhibits 23

Figure D1. The frequency distribution of food item quotations per country 23

Table D1. The number of food products priced in each ICP country in the study sample, for all foods and ELD food groups........ 24

Table D2. Medians of country-level shares of food items priced relative to the full global list of ICP foods, by region and income level ... 29

Figure D2. Local polynomial plots of country-level ELD food group costs against the share of food group items priced in each country ... 30

Table D3. Summary statistics of country-levels shares of food items priced relative to the full global list of ICP food ... 33

Table D4. Gauging the potential extent of urban bias in ICP national price surveys ... 34

Figure D3. Partial local polynomial plots of country-level ELD food group costs against the extent of urban bias in food surveys ... 35

Table D5. Robust regressions of ELD food group costs against "bias" measures in 141 countries, with additional controls ... 38

Adjusting ELD costs for low product coverage ... 39

Table D6. Population-weighted mean ELD and food group costs with and without adjustments for product coverage bias by World Bank regions (25th percentile floor method) ... 40

Appendix E. Varying physical activity levels and calorie requirements after applying a sedentary lifestyle assumption ... 41

Table E1. Revised estimates of the number and share of the population in 137 countries that are poor according to an EAT-*Lancet* reference diet food poverty line and country-specific non-food poverty lines assuming sedentary activity levels by country income level and geographic region ... 41

Appendix A. Additional details about the EAT-Lancet diet and International Comparison Program (ICP) data

Table A1. Composition of the EAT-*Lancet* reference diet (ELD), by food group

**EAT-*Lancet* groups**	**Serving (kcal/d)**	**Functional category**	**Serving (kcal/d)**	**New functional categorization**	**Serving (kcal/d)**
Rice, wheat, corn, and other	811	Rice, wheat, corn, and other	811	Rice, wheat, corn, and other	811
Potatoes and cassava	39	Potatoes and cassava	39	Potatoes and cassava	39
Dark green vegetables	23	Dark green vegetables	23		
Red and orange vegetables	30	Red and orange vegetables	30	Red and orange vegetables	30
Other vegetables	25	Other vegetables	25	Other vegetables, including Dark green	48
All fruits	126	All fruits	126	All fruits	126
Whole milk or equivalents	153	Whole milk or equivalents	153	Whole milk or equivalents	153
Beef and lamb	15	Beef, lamb and pork	30	Beef, lamb and pork	30
Pork	15				
Chicken and other poultry	62	Poultry, eggs and fish	121	Poultry, eggs and fish	121
Eggs	19				
Fish	40				
Dry beans, lentils, and peas	172	Legumes, nuts & soy foods	575	Legumes, nuts & soy foods	575
Soy foods	112				
Peanuts	142				
Tree nuts	149				
Palm oil	60	Palm oil	60	Oils & fats	450
Unsaturated oils	354	Unsaturated oils	354		
Dairy fats	0	Dairy fats	0		
Lard or tallow	36	Lard or tallow	36		
All sweeteners	120	All sweeteners	120	All sweeteners	120
**Total**	**2,503**	**Total**	**2,503**	**Total**	**2,503**

Notes: Data are daily servings in kcal. The healthy reference diet described by Willett et al (2019) is reported in the first column ('EAT-*Lancet* groups'). The middle column (‘Functional food groups') provides the aggregations of the EAT-*Lancet* food groups used in Hirvonen et al. (2020) and the last column the aggregations used in this paper.

Table A2. The 137 ICP countries included in the main analyses

Name	Region	Income level	Population in millions
Australia	East Asia and Pacific	High income	22.3
Austria	Europe and Central Asia	High income	8.4
Belgium	Europe and Central Asia	High income	11.0
Canada	North America	High income	34.3
Chile	Latin America and Caribbean	High income	17.3
Croatia	Europe and Central Asia	High income	4.3
Cyprus	Europe and Central Asia	High income	0.9
Czech Republic	Europe and Central Asia	High income	10.5
Denmark	Europe and Central Asia	High income	5.6
Estonia	Europe and Central Asia	High income	1.3
Finland	Europe and Central Asia	High income	5.4
France	Europe and Central Asia	High income	65.3
Germany	Europe and Central Asia	High income	80.3
Greece	Europe and Central Asia	High income	11.1
Hungary	Europe and Central Asia	High income	10.0
Iceland	Europe and Central Asia	High income	0.3
Ireland	Europe and Central Asia	High income	4.6
Israel	Middle East and North Africa	High income	7.8
Italy	Europe and Central Asia	High income	60.1
Japan	East Asia and Pacific	High income	127.8
Korea, Rep.	East Asia and Pacific	High income	49.9
Latvia	Europe and Central Asia	High income	2.1
Lithuania	Europe and Central Asia	High income	3.0
Luxembourg	Europe and Central Asia	High income	0.5
Malta	Middle East and North Africa	High income	0.4
Netherlands	Europe and Central Asia	High income	16.7
Norway	Europe and Central Asia	High income	5.0
Poland	Europe and Central Asia	High income	38.5
Portugal	Europe and Central Asia	High income	10.6
Slovakia	Europe and Central Asia	High income	5.4
Slovenia	Europe and Central Asia	High income	2.1
Spain	Europe and Central Asia	High income	46.7
Sweden	Europe and Central Asia	High income	9.4
Switzerland	Europe and Central Asia	High income	7.9
United Kingdom	Europe and Central Asia	High income	63.3
United States	North America	High income	312.0
Panama	Latin America and Caribbean	High income	3.7
Uruguay	Latin America and Caribbean	High income	3.4
United Arab Emirates	Middle East and North Africa	High income	8.7
Trinidad and Tobago	Latin America and Caribbean	High income	1.3

Name	Region	Income level	Population in millions
Algeria	Middle East and North Africa	Upper middle income	36.7
Botswana	Sub-Saharan Africa	Upper middle income	2.0
Gabon	Sub-Saharan Africa	Upper middle income	1.7
Mauritius	Sub-Saharan Africa	Upper middle income	1.3
Namibia	Sub-Saharan Africa	Upper middle income	2.2
South Africa	Sub-Saharan Africa	Upper middle income	52.0
China	East Asia and Pacific	Upper middle income	1344.1
Fiji	East Asia and Pacific	Upper middle income	0.9
Malaysia	East Asia and Pacific	Upper middle income	29.1
Maldives	South Asia	Upper middle income	0.4
Sri Lanka	South Asia	Upper middle income	20.2
Thailand	East Asia and Pacific	Upper middle income	66.2
Armenia	Europe and Central Asia	Upper middle income	3.0
Azerbaijan	Europe and Central Asia	Upper middle income	9.1
Belarus	Europe and Central Asia	Upper middle income	9.5
Kazakhstan	Europe and Central Asia	Upper middle income	16.6
Albania	Europe and Central Asia	Upper middle income	2.9
Bosnia and Herzegovina	Europe and Central Asia	Upper middle income	3.7
Bulgaria	Europe and Central Asia	Upper middle income	7.3
Macedonia, FYR	Europe and Central Asia	Upper middle income	2.1
Mexico	Latin America and Caribbean	Upper middle income	115.5
Montenegro	Europe and Central Asia	Upper middle income	0.6
Romania	Europe and Central Asia	Upper middle income	20.1
Russian Federation	Europe and Central Asia	Upper middle income	143.0
Serbia	Europe and Central Asia	Upper middle income	7.2
Turkey	Europe and Central Asia	Upper middle income	74.2
Brazil	Latin America and Caribbean	Upper middle income	197.5
Colombia	Latin America and Caribbean	Upper middle income	46.0
Costa Rica	Latin America and Caribbean	Upper middle income	4.6
Dominican Republic	Latin America and Caribbean	Upper middle income	9.8
Ecuador	Latin America and Caribbean	Upper middle income	15.2
Paraguay	Latin America and Caribbean	Upper middle income	6.3
Peru	Latin America and Caribbean	Upper middle income	29.3
Iraq	Middle East and North Africa	Upper middle income	33.3
Jordan	Middle East and North Africa	Upper middle income	7.0
Jamaica	Latin America and Caribbean	Upper middle income	2.8
Angola	Sub-Saharan Africa	Lower middle income	24.2
Cameroon	Sub-Saharan Africa	Lower middle income	20.9
Cape Verde	Sub-Saharan Africa	Lower middle income	0.5
Comoros	Sub-Saharan Africa	Lower middle income	0.7
Congo, Rep.	Sub-Saharan Africa	Lower middle income	4.4
Côte d'Ivoire	Sub-Saharan Africa	Lower middle income	21.0
Djibouti	Middle East and North Africa	Lower middle income	0.9

Name	Region	Income level	Population in millions
Egypt, Arab Rep.	Middle East and North Africa	Lower middle income	80.5
Ghana	Sub-Saharan Africa	Lower middle income	25.4
Kenya	Sub-Saharan Africa	Lower middle income	43.2
Lesotho	Sub-Saharan Africa	Lower middle income	2.0
Mauritania	Sub-Saharan Africa	Lower middle income	3.6
Morocco	Middle East and North Africa	Lower middle income	32.6
Nigeria	Sub-Saharan Africa	Lower middle income	162.8
São Tomé and Principe	Sub-Saharan Africa	Lower middle income	0.2
Senegal	Sub-Saharan Africa	Lower middle income	13.0
Sudan	Sub-Saharan Africa	Lower middle income	34.0
Swaziland	Sub-Saharan Africa	Lower middle income	1.1
Tunisia	Middle East and North Africa	Lower middle income	10.7
Zambia	Sub-Saharan Africa	Lower middle income	14.0
Zimbabwe	Sub-Saharan Africa	Lower middle income	12.9
Bangladesh	South Asia	Lower middle income	149.7
Bhutan	South Asia	Lower middle income	0.7
India	South Asia	Lower middle income	1216.1
Indonesia	East Asia and Pacific	Lower middle income	242.0
Lao PDR	East Asia and Pacific	Lower middle income	6.1
Mongolia	East Asia and Pacific	Lower middle income	2.8
Myanmar	East Asia and Pacific	Lower middle income	49.7
Pakistan	South Asia	Lower middle income	177.1
Philippines	East Asia and Pacific	Lower middle income	94.2
Vietnam	East Asia and Pacific	Lower middle income	88.1
Kyrgyzstan	Europe and Central Asia	Lower middle income	5.3
Moldova	Europe and Central Asia	Lower middle income	3.6
Bolivia	Latin America and Caribbean	Lower middle income	10.2
El Salvador	Latin America and Caribbean	Lower middle income	6.2
Honduras	Latin America and Caribbean	Lower middle income	8.5
Nicaragua	Latin America and Caribbean	Lower middle income	5.9
Benin	Sub-Saharan Africa	Low income	9.5
Burkina Faso	Sub-Saharan Africa	Low income	16.1
Burundi	Sub-Saharan Africa	Low income	9.0
Central African Republic	Sub-Saharan Africa	Low income	4.4
Chad	Sub-Saharan Africa	Low income	12.4
Congo, Dem. Rep.	Sub-Saharan Africa	Low income	66.8
Ethiopia	Sub-Saharan Africa	Low income	90.1
Gambia, The	Sub-Saharan Africa	Low income	1.8
Guinea	Sub-Saharan Africa	Low income	10.4
Guinea-Bissau	Sub-Saharan Africa	Low income	1.6
Liberia	Sub-Saharan Africa	Low income	4.0
Madagascar	Sub-Saharan Africa	Low income	21.7
Malawi	Sub-Saharan Africa	Low income	15.0

Name	Region	Income level	Population in millions
Mali	Sub-Saharan Africa	Low income	15.5
Mozambique	Sub-Saharan Africa	Low income	24.2
Niger	Sub-Saharan Africa	Low income	17.1
Rwanda	Sub-Saharan Africa	Low income	10.3
Sierra Leone	Sub-Saharan Africa	Low income	6.6
Tanzania	Sub-Saharan Africa	Low income	45.7
Togo	Sub-Saharan Africa	Low income	6.6
Uganda	Sub-Saharan Africa	Low income	33.5
Nepal	South Asia	Low income	26.5
Tajikistan	Europe and Central Asia	Low income	7.7
Haiti	Latin America and Caribbean	Low income	10.1

Table A3. The ICP countries not included in the main analyses

Name	Region	Income level	Population in millions
Antigua and Barbuda	Latin America and Caribbean	High income	0.1
Aruba	Latin America and Caribbean	High income	0.1
Bahamas, The	Latin America and Caribbean	High income	0.4
Bahrain	Middle East and North Africa	High income	1.5
Barbados	Latin America and Caribbean	High income	0.3
Bermuda	North America	High income	0.1
British Virgin Islands	Latin America and Caribbean	High income	0.0
Brunei Darussalam	East Asia and Pacific	High income	0.4
Cayman Islands	Latin America and Caribbean	High income	0.1
Curacao	Latin America and Caribbean	High income	0.2
French Polynesia	East Asia and Pacific	High income	0.3
Guam	East Asia and Pacific	High income	0.2
Hong Kong SAR, China	East Asia and Pacific	High income	7.4
Kuwait	Middle East and North Africa	High income	4.1
Macao SAR, China	East Asia and Pacific	High income	0.6
Nauru	East Asia and Pacific	High income	0.0
New Caledonia	East Asia and Pacific	High income	0.3
New Zealand	East Asia and Pacific	High income	4.8
Northern Mariana Islands	East Asia and Pacific	High income	0.1
Oman	Middle East and North Africa	High income	4.5
Palau	East Asia and Pacific	High income	0.0
Puerto Rico	Latin America and Caribbean	High income	3.3
Qatar	Middle East and North Africa	High income	2.7
San Marino	Europe and Central Asia	High income	0.0
Saudi Arabia	Middle East and North Africa	High income	34.2
Seychelles	Sub-Saharan Africa	High income	0.1
Singapore	East Asia and Pacific	High income	5.6
Sint Maarten (Dutch part)	Latin America and Caribbean	High income	0.0
St Kitts and Nevis	Latin America and Caribbean	High income	0.0
Turks and Caicos Islands	Latin America and Caribbean	High income	0.0
American Samoa	East Asia and Pacific	Upper middle income	0.0
Argentina	Latin America and Caribbean	Upper middle income	44.0
Belize	Latin America and Caribbean	Upper middle income	0.4
Cuba	Latin America and Caribbean	Upper middle income	11.3
Dominica	Latin America and Caribbean	Upper middle income	0.1
Equatorial Guinea	Sub-Saharan Africa	Upper middle income	1.5
Georgia	Europe and Central Asia	Upper middle income	3.7
Grenada	Latin America and Caribbean	Upper middle income	0.1
Guatemala	Latin America and Caribbean	Upper middle income	16.1
Guyana	Latin America and Caribbean	Upper middle income	0.8
Iran, Islamic Rep	Middle East and North Africa	Upper middle income	84.5

Name	Region	Income level	Population in millions
Lebanon	Middle East and North Africa	Upper middle income	6.1
Libya	Middle East and North Africa	Upper middle income	6.4
Marshall Islands	East Asia and Pacific	Upper middle income	0.0
Samoa	East Asia and Pacific	Upper middle income	0.2
St Lucia	Latin America and Caribbean	Upper middle income	0.2
St Vincent and the Grenadines	Latin America and Caribbean	Upper middle income	0.1
Suriname	Latin America and Caribbean	Upper middle income	0.6
Tonga	East Asia and Pacific	Upper middle income	0.1
Turkmenistan	Europe and Central Asia	Upper middle income	6.0
Tuvalu	East Asia and Pacific	Upper middle income	0.0
Venezuela	Latin America and Caribbean	Upper middle income	30.6
Cambodia	East Asia and Pacific	Lower middle income	15.8
Kiribati	East Asia and Pacific	Lower middle income	0.1
Micronesia, Fed Sts	East Asia and Pacific	Lower middle income	0.1
Papua New Guinea	East Asia and Pacific	Lower middle income	9.1
Solomon Islands	East Asia and Pacific	Lower middle income	0.6
Timor-Leste	East Asia and Pacific	Lower middle income	1.2
Ukraine	Europe and Central Asia	Lower middle income	44.8
Uzbekistan	Europe and Central Asia	Lower middle income	32.4
Vanuatu	East Asia and Pacific	Lower middle income	0.3
West Bank and Gaza	Middle East and North Africa	Lower middle income	4.5
Afghanistan	South Asia	Low income	35.6
Eritrea	Sub-Saharan Africa	Low income	3.4
Somalia	Sub-Saharan Africa	Low income	14.9
South Sudan	Sub-Saharan Africa	Low income	10.7
Yemen, Rep	Middle East and North Africa	Low income	30.0

Table A4. Least ELD cost based on ICP-2011 in 137 countries, based on 15 food groups versus 11 food groups

	N	1. ELD cost, 15 food groups (2011 $PPP)	2. ELD cost, 11 food groups (2011 $PPP)	3. Difference (1.-2.) (2011 $PPP)
**Global**	**137**	**$2.83**	**$2.60**	**$0.23**
**By income level:**				
High income	39	$2.69	$2.32	$0.36
Upper middle	36	$3.11	$2.92	$0.18
Lower middle	36	$3.00	$2.82	$0.18
Low income	26	$2.43	$2.28	$0.15
**By geographic region:**				
East Asia & Pacific	13	$3.37	$3.01	$0.36
Europe & Central Asia	44	$2.85	$2.56	$0.30
Latin America & Caribbean	18	$3.31	$3.13	$0.18
Middle East & North Africa	10	$2.71	$2.50	$0.21
North America	2	$2.65	$2.65	$0.00
South Asia	7	$2.80	$2.71	$0.09
Sub-Saharan Africa	43	$2.48	$2.31	$0.18

Note: This table reports the ELD-cost estimates in 2011 PPP dollars based on ICP-2011 data. Column 1 is based on the same food grouping used by Hirvonen et al (2020). Column 2 is based on the same ICP data after combining 'Dark green vegetables' food group with 'Other vegetables' food group, and merging Palm Oil, Lard/Tallow and Unsaturated oils into Oils & fats group. The country income level grouping is based on 2019 World Bank classification (same as in Hirvonen et al 2020) and therefore differs slightly with the more recent classification used elsewhere in this paper.

Appendix B. Additional details on the demographic scaling factor

Figure B1. Differences in the population “pyramids” between China and India

Panel A: China **Panel B: India**

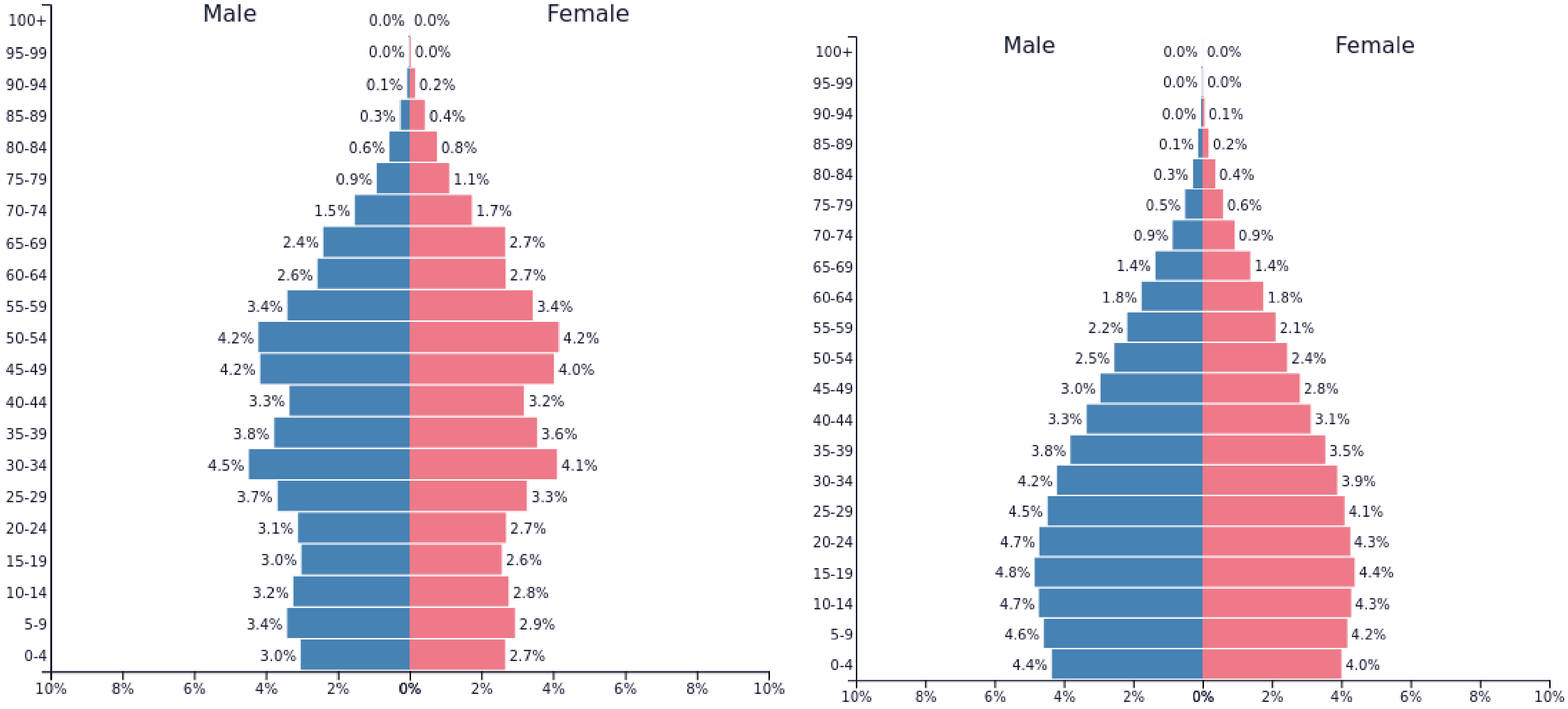

Source: https://www.populationpyramid.net/

Table B1. Estimated human energy requirements

Age category	Males		Females	
	kcal	per adult female	kcal	per adult female
age 0-4	1,169	0.47	1,075	0.43
age 5-9	1,710	0.68	1,570	0.63
age 10-14	2,565	1.03	2,250	0.90
age 15-19	3,300	1.32	2,500	1.00
age 20-29	2,950	1.18	2,350	0.94
age 30-59	2,500	1.00	2,500 (*)	1.00
age 60-	2,450	0.98	2,350	0.94

Note: Constructed using FAO et al. (2004) estimates for human energy requirement, except (*) from Willett et al. (2019). 'Adult female' refers to females age 30-59.

Figure B2. Distributions of the demographic scaling factors across the 137 countries in our data

A. 'Age-sex' scaling factor

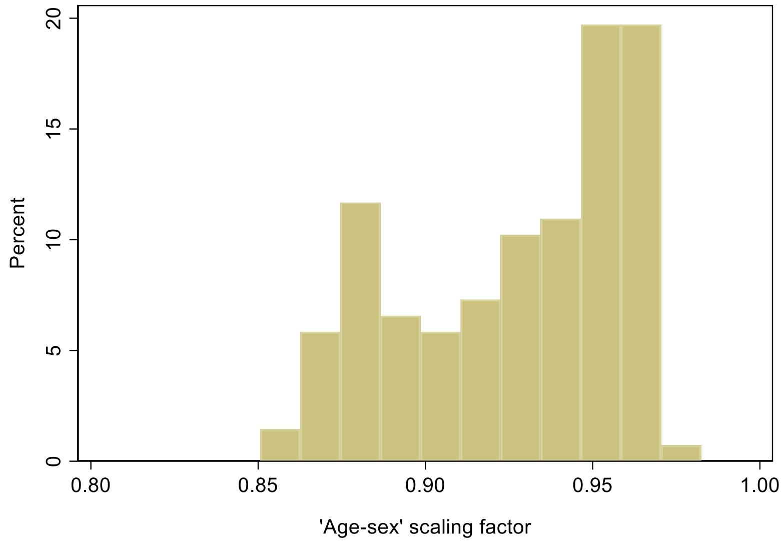

B. ‘Pregnancy-lactation' scaling factor

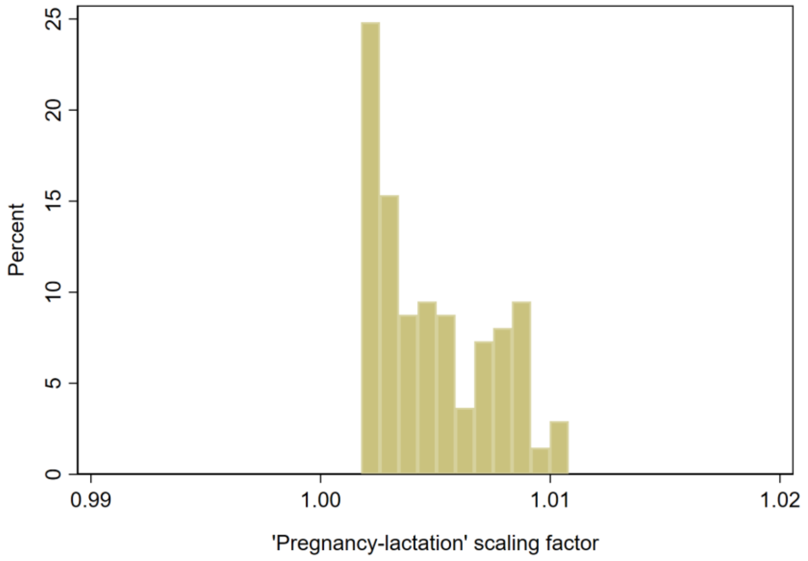

Source: Authors’ estimate. The scaling factors are described in the main text. 'Age-sex' scaling factor is an adjustment for average calorie requirements of a population relative to the 2,500-calorie reference intake of a 30-year-old woman who is not pregnant or lactating. ‘Pregnancy-lactation' scaling factor adjusts for the share of population that is pregnant or lactating taking account the increased energy needs during pregnancy and lactation.

Table B2. Estimated demographic scaling factor, by country

Country	'Age-sex' scaling factor	‘Pregnancy-lactation' scaling factor
Angola	0.863	1.010
Albania	0.964	1.003
United Arab Emirates	0.982	1.003
Armenia	0.948	1.003
Australia	0.951	1.003
Austria	0.963	1.002
Azerbaijan	0.944	1.004
Burundi	0.869	1.009
Belgium	0.953	1.002
Benin	0.881	1.009
Burkina Faso	0.873	1.009
Bangladesh	0.936	1.004
Bulgaria	0.958	1.002
Bosnia and Herzegovina	0.963	1.002
Belarus	0.954	1.003
Bolivia	0.922	1.005
Brazil	0.952	1.003
Bhutan	0.946	1.004
Botswana	0.909	1.006
Central African Republic	0.880	1.010
Canada	0.959	1.002
Switzerland	0.960	1.002
Chile	0.952	1.003
China	0.959	1.003
Côte d'Ivoire	0.886	1.008
Cameroon	0.881	1.009
Congo, Dem. Rep.	0.865	1.010
Congo, Rep.	0.882	1.008
Colombia	0.947	1.004
Comoros	0.893	1.007
Cabo Verde	0.932	1.004
Costa Rica	0.951	1.003
Cyprus	0.964	1.003
Czech Republic	0.956	1.002
Germany	0.964	1.002
Djibouti	0.931	1.005
Denmark	0.958	1.002
Dominican Republic	0.932	1.005
Algeria	0.920	1.006

Country	'Age-sex' scaling factor	'Pregnancy-lactation' scaling factor
Ecuador	0.931	1.004
Egypt, Arab Rep.	0.910	1.006
Spain	0.958	1.002
Estonia	0.953	1.002
Ethiopia	0.890	1.008
Finland	0.954	1.002
Fiji	0.923	1.005
France	0.950	1.003
Gabon	0.900	1.007
United Kingdom	0.951	1.003
Ghana	0.901	1.007
Guinea	0.878	1.009
Gambia, The	0.875	1.008
Guinea-Bissau	0.881	1.008
Greece	0.960	1.002
Honduras	0.924	1.005
Croatia	0.960	1.002
Haiti	0.916	1.006
Hungary	0.962	1.002
Indonesia	0.935	1.004
India	0.938	1.004
Ireland	0.939	1.003
Iraq	0.897	1.007
Iceland	0.948	1.003
Israel	0.923	1.005
Italy	0.961	1.002
Jamaica	0.946	1.003
Jordan	0.910	1.005
Japan	0.960	1.002
Kazakhstan	0.921	1.005
Kenya	0.893	1.007
Kyrgyz Republic	0.914	1.006
Korea, Rep.	0.970	1.002
Lao PDR	0.920	1.006
Liberia	0.886	1.008
Sri Lanka	0.937	1.004
Lesotho	0.919	1.007
Lithuania	0.961	1.002
Luxembourg	0.960	1.002
Latvia	0.956	1.003
Morocco	0.931	1.005
Moldova	0.964	1.003

Country	'Age-sex' scaling factor	‘Pregnancy-lactation' scaling factor
Madagascar	0.890	1.008
Maldives	0.963	1.004
Mexico	0.935	1.004
North Macedonia	0.961	1.003
Mali	0.862	1.010
Malta	0.963	1.002
Myanmar	0.938	1.004
Montenegro	0.955	1.003
Mongolia	0.920	1.006
Mozambique	0.874	1.009
Mauritania	0.890	1.008
Mauritius	0.960	1.002
Malawi	0.877	1.008
Malaysia	0.947	1.004
Namibia	0.903	1.007
Niger	0.851	1.011
Nigeria	0.876	1.009
Nicaragua	0.926	1.005
Netherlands	0.958	1.002
Norway	0.954	1.003
Nepal	0.923	1.005
Pakistan	0.911	1.007
Panama	0.931	1.005
Peru	0.934	1.004
Philippines	0.922	1.005
Poland	0.961	1.002
Portugal	0.962	1.002
Paraguay	0.930	1.005
Romania	0.959	1.002
Russian Federation	0.951	1.003
Rwanda	0.892	1.007
Sudan	0.890	1.008
Senegal	0.878	1.008
Sierra Leone	0.888	1.008
El Salvador	0.936	1.004
Serbia	0.960	1.002
São Tomé and Principe	0.883	1.007
Slovak Republic	0.960	1.002
Slovenia	0.957	1.002
Sweden	0.951	1.003
Eswatini	0.903	1.006
Chad	0.864	1.011

Country	'Age-sex' scaling factor	‘Pregnancy-lactation' scaling factor
Togo	0.885	1.008
Thailand	0.959	1.002
Tajikistan	0.903	1.007
Trinidad and Tobago	0.948	1.003
Tunisia	0.938	1.004
Turkey	0.939	1.004
Tanzania	0.875	1.009
Uganda	0.864	1.009
Uruguay	0.948	1.003
United States	0.944	1.003
Vietnam	0.945	1.004
South Africa	0.927	1.005
Zambia	0.873	1.009
Zimbabwe	0.881	1.008

Appendix C. Additional details about non-food budgets

Allen (2017) estimated minimum non-food budget by calculating the minimum cost of housing, fuel, lighting, clothing, and soap. Below we describe the data and methods used to estimate the cost for each of these cost-components.

Housing cost needs

- **Method:** Allen sets the housing needs at 3 square meters per person.
- **Data:** The ICP data provides rental amounts per square meter for different types of apartments (studios, 1–3-bedroom apartments, detached & non-detached houses). We can use these ICP entries to calculate the cheapest rental price per square meter.
- **Data gaps:** These ICP rental price data are missing for all OECD countries. To address this, we used "2017 Current Market Rents from Surveys through Estate Agencies", a report compiled by International Service for Remunerations and Pensions (ISRP) at the OECD and Eurostat. The report covers 5 different types of dwellings: 1–3-bedroom apartments, detached & non-detached houses. The limitation of these data is that they are not nationally representative (instead cover only 1-2 major cities per country). Consequently, the rental prices are likely to be higher than national averages. In addition, only the range of square meters of different apartment types is provided. We used the upper range of the square meter estimate to calculate the rental prices per square meters.

Fuel cost needs for heating & cooking, and lighting

- **Method:** These requirements depend on climate (temperature). The external temperature is measured by the "heating-degree days," that is, the sum over the year of the difference between the desired internal temperature and the external temperature. Allen's excel files provided in Supplementary data provide formulas that calculate the heating and cooking needs based on heating degree days. In hot climates, the minimum is set at 1.6 million British Thermal Units (BTUs) based on the energy poverty line of the Millennium Development Goals.
- **Data:** Allen used heating degree-days calculated at half hour intervals over five years for most airports and weather stations in the world. We used population-weighted degree days (average over 1964-2013), provided by Atalla et al. (2018). ICP provides cost estimates for different energy types (electricity, propane, gas, charcoal, and kerosene). After converting these energy sources to BTUs, we can use these ICP entries to calculate the cost of cheapest BTU.

- **Data gaps:** Degree Day data are missing for approximately 20 countries; these countries are mostly small in size. For a handful of European countries, we can obtain a heating degree-day estimate from Eurostat website. The degree days for the remaining countries needed to be hand-picked from academic publications. Additionally, the energy prices in the ICP data are sometimes missing. We replaced the missing prices using price estimates from neighboring countries.

Clothing cost needs

- **Method:** These requirements also depend on climate (temperature), i.e., "heating-degree days". Allen's excel files provided in Supplementary data provide formulas that calculate the clothing needs based on heating degree days. As before, in hot climates, the minimum is set at 1.6 million British Thermal Units (BTUs) based on the energy poverty line of the Millennium Development Goals. In hot climates, Allen's formula sets the minimum cloth need at 19 meters per person per year.
- **Data:** Allen used heating degree-days calculated at half hour intervals over five years for most airports and weather stations in the world. We used population-weighted degree days (average over 1964-2013), provided by Atalla et al. (2018). ICP provides cost estimates for different cloth types (by running meter).
- **Data gaps:** Some missing observations for cloth prices in the ICP data. Solution: Imputed using data from neighboring countries.

Soap cost needs

- **Method:** Allen set the soap requirement at 1.3 kg per year (25 g per week) per person.
- **Data:** Following Allen, we only consider bar soap (not liquid soaps) and then calculate the daily per person cost based on the soap needs defined above.
- **Data gaps:** Soap prices are missing for about 10 percent of the countries, out of which many are in Europe. Solution: Imputed using data from neighboring countries.

Figure C1. Association between cost of the food and non-food budgets

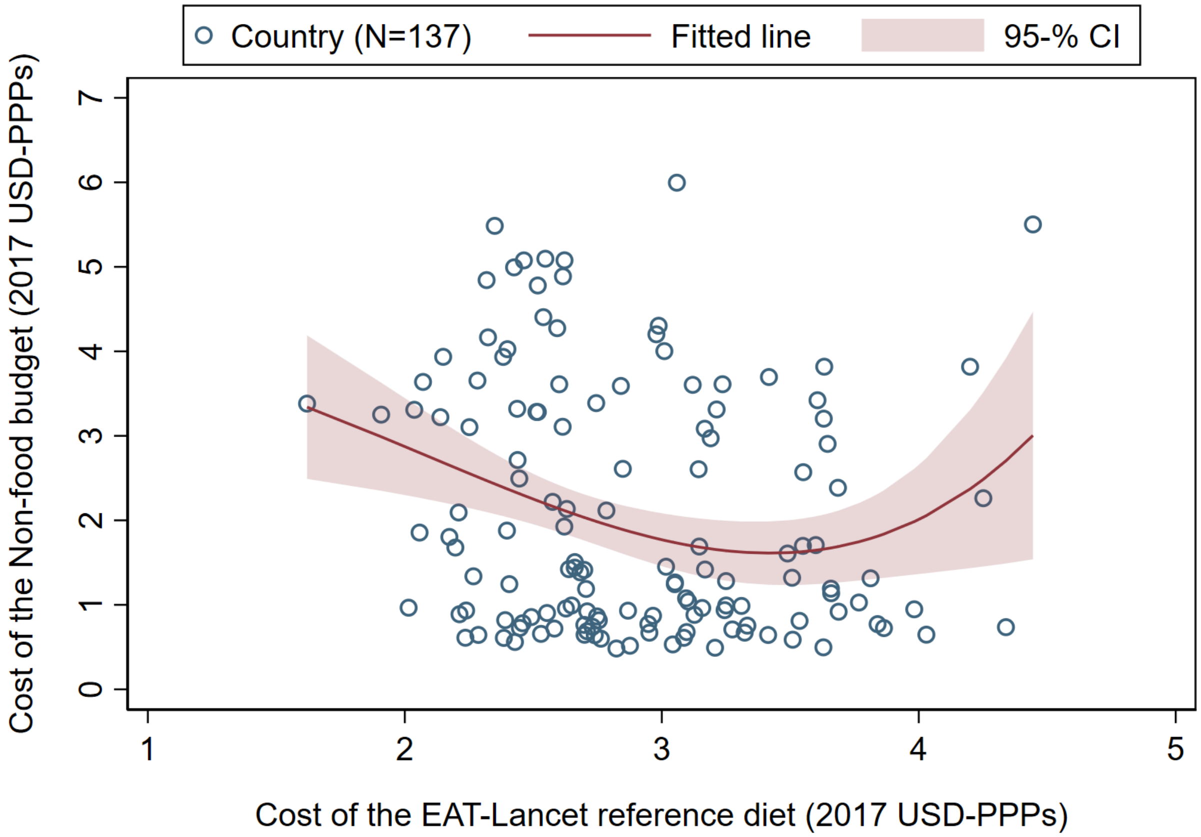

Note: N = 137 countries. Scatter plot with a fitted line based on fractional-polynomial prediction. CI = Confidence interval.

Appendix D. Correcting for urban bias and short product lists in the ICP data, additional exhibits

Figure D1. The frequency distribution of food item quotations per country

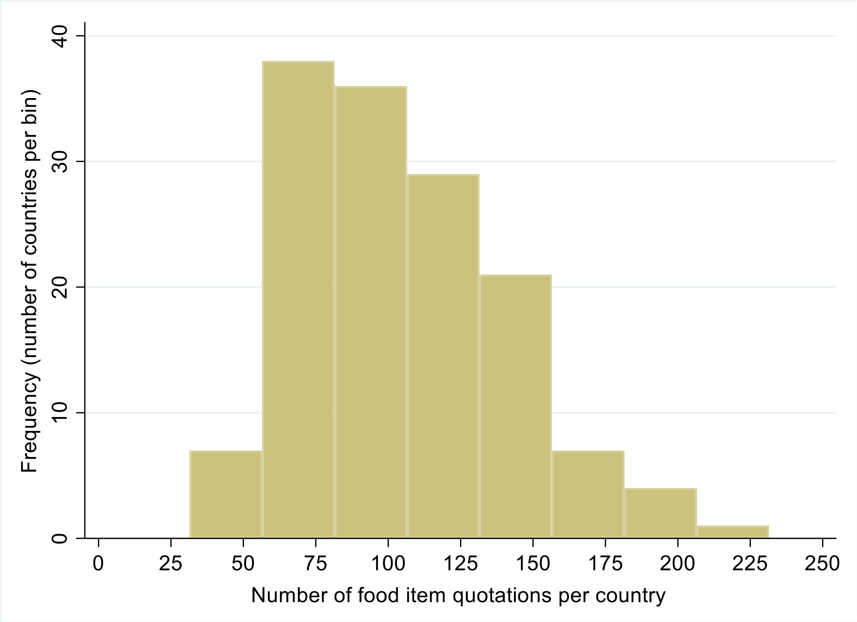

Notes. Results estimated by the authors for 141 countries from 2017 ICP national average prices as described in World Bank (2020) and the main text.

Table D1. The number of food products priced in each ICP country in the study sample, for all foods and ELD food groups

	All foods	Starchy staples	Oils, fats	Sugar	Beans, nuts, seeds	Vege- tables	Fruit	Dairy	Red meat	Poultry & fish
Albania	78	20	3	2	4	8	9	7	9	15
Algeria	91	24	3	5	5	9	7	15	13	16
Angola	69	16	3	3	2	7	6	7	11	14
Argentina	67	16	3	2	1	9	9	8	9	9
Armenia	169	43	5	7	6	20	22	29	24	22
Australia	80	18	4	2	4	9	9	10	10	15
Austria	80	21	4	2	3	9	9	9	11	12
Bahamas, The	47	15	1	1	1	6	5	6	5	7
Bahrain	130	26	4	3	9	14	23	12	18	19
Bangladesh	171	50	9	4	10	13	12	12	21	39
Belarus	165	40	4	6	7	19	20	25	24	26
Belgium	77	21	4	2	3	9	9	7	10	10
Benin	128	33	6	5	4	9	12	15	22	28
Bhutan	92	26	1	3	5	14	13	7	7	9
Bolivia	56	13	3	1	2	8	8	4	8	5
Bosnia Herzegovina	76	19	3	1	4	9	9	8	7	15
Botswana	100	23	5	4	6	9	10	14	19	15
Brazil	78	17	3	1	3	9	10	8	11	15
Brunei	144	35	7	3	10	16	13	10	10	34
Bulgaria	87	22	4	2	4	9	9	10	11	17
Burkina Faso	128	32	7	6	5	9	13	16	22	25
Burundi	116	28	6	4	6	9	13	12	21	20
Cambodia	141	36	7	4	4	15	12	10	20	28
Cameroon	131	32	7	5	6	9	13	16	22	28
Central African rep.	104	28	6	5	6	8	8	15	19	16
Chad	131	33	7	6	5	9	12	15	22	28
Chile	75	18	4	1	4	9	8	8	9	13
China	194	55	8	4	6	16	15	14	32	42
Colombia	67	16	4	2	3	7	8	4	10	10
Comoros	72	18	5	5	5	1	6	15	9	22

	All foods	Starchy staples	Oils, fats	Sugar	Beans, nuts, seeds	Vege-tables	Fruit	Dairy	Red meat	Poultry & fish
Congo, Dem. Rep.	129	31	7	5	6	9	13	16	22	27
Congo, Rep.	133	33	7	5	6	9	13	16	22	29
Costa Rica	53	11	4	2	2	6	5	7	8	9
Croatia	84	22	4	2	3	9	9	9	10	16
Cyprus	84	22	4	2	4	8	9	10	11	16
Czech Republic	81	20	3	1	3	9	9	10	10	17
Côte d'Ivoire	130	33	7	6	5	9	12	16	21	28
Denmark	82	21	4	2	4	8	9	7	10	16
Djibouti	102	28	4	5	5	9	9	15	18	15
Dominican Rep.	96	22	4	2	6	11	11	10	15	14
Ecuador	67	17	3	2	3	8	10	5	7	9
Egypt, Arab Rep.	106	25	4	4	5	9	12	16	16	22
El Salvador	44	11	1	1	0	5	2	3	10	9
Equatorial Guinea	134	33	7	6	6	9	13	16	22	29
Estonia	79	20	4	2	3	9	8	10	11	13
Ethiopia	123	33	7	5	5	9	12	14	20	23
Fiji	82	21	2	2	5	7	9	5	14	15
Finland	71	20	3	2	2	8	8	9	9	11
France	83	22	3	2	4	8	8	9	11	17
Gabon	105	21	7	5	6	9	11	13	14	23
Gambia, The	133	33	7	6	6	9	13	16	22	28
Germany	77	21	4	2	3	8	9	10	10	12
Ghana	130	31	6	6	6	9	13	15	22	28
Greece	85	22	4	2	4	9	9	9	11	15
Guinea	118	27	7	5	6	9	13	16	17	25
Guinea-Bissau	133	33	7	5	6	9	13	16	22	29
Guyana	85	23	4	2	6	8	5	10	16	13
Haiti	61	14	2	2	3	8	9	7	7	8
Honduras	56	13	2	1	3	6	10	6	8	7
Hungary	80	21	3	2	4	9	7	9	11	14
Iceland	72	18	3	1	3	8	9	8	10	12
India	208	57	9	4	11	17	16	15	33	44
Indonesia	155	37	6	4	5	15	16	12	19	38

	All foods	Starchy staples	Oils, fats	Sugar	Beans, nuts, seeds	Vege-tables	Fruit	Dairy	Red meat	Poultry & fish
Iraq	153	32	4	3	12	16	25	13	17	28
Ireland	79	20	4	2	4	8	8	9	10	15
Israel	83	19	4	2	4	9	9	9	11	16
Italy	84	21	4	2	4	9	9	10	11	15
Jamaica	56	12	1	2	1	9	4	5	8	10
Japan	56	11	3	1	3	8	7	7	7	8
Jordan	155	32	4	3	12	16	25	13	19	28
Kazakhstan	204	53	6	7	9	24	25	28	26	30
Kenya	112	28	7	5	6	9	13	15	15	20
Korea, Rep.	67	13	4	1	3	8	9	8	8	13
Kuwait	148	33	4	3	12	15	24	13	15	27
Kyrgyz Republic	171	44	5	6	9	16	22	27	30	23
Lao PDR	139	35	1	3	4	14	15	10	29	24
Latvia	83	21	4	2	4	9	9	10	10	15
Lesotho	99	25	5	3	4	9	10	14	21	13
Liberia	109	30	5	5	6	9	10	15	16	19
Lithuania	83	22	4	2	4	9	9	10	10	14
Luxembourg	84	22	4	2	3	9	9	10	11	15
Madagascar	100	26	4	4	4	9	11	12	16	17
Malawi	123	33	5	6	5	9	13	16	22	21
Malaysia	144	30	6	3	7	17	13	9	17	34
Maldives	57	13	5	2	2	10	9	8	0	6
Mali	131	32	7	6	6	9	13	16	22	27
Malta	85	22	4	2	4	9	9	10	11	15
Mauritania	89	28	5	4	5	8	10	15	10	11
Mauritius	117	28	5	5	6	9	13	15	21	21
Mexico	78	19	3	2	4	9	8	10	10	14
Moldova	173	46	4	5	8	17	20	29	33	23
Mongolia	126	39	7	4	6	15	13	10	13	14
Morocco	120	30	5	5	6	9	13	16	20	23
Mozambique	131	33	6	6	6	9	13	16	22	27
Myanmar	192	55	9	4	9	17	16	15	28	37
Namibia	105	30	4	3	4	9	12	14	18	16

	All foods	Starchy staples	Oils, fats	Sugar	Beans, nuts, seeds	Vege-tables	Fruit	Dairy	Red meat	Poultry & fish
Nepal	115	38	4	2	9	13	11	9	8	17
Netherlands	82	22	4	2	4	9	9	9	10	13
New Zealand	60	15	3	2	3	7	6	8	8	9
Nicaragua	69	15	3	1	2	11	10	8	7	9
Niger	125	30	6	5	5	9	12	16	22	27
Nigeria	130	32	7	6	6	9	13	16	21	27
North Macedonia	71	19	2	1	3	8	7	8	10	13
Norway	72	20	3	2	2	8	8	9	9	12
Oman	153	32	4	3	12	16	24	13	19	27
Pakistan	187	55	7	4	11	16	14	15	22	42
Panama	70	18	4	2	2	8	10	9	8	10
Paraguay	71	18	3	1	2	10	9	9	9	9
Peru	93	23	4	2	6	11	10	9	13	13
Philippines	169	36	8	4	8	16	13	12	27	41
Poland	81	19	4	2	4	9	9	10	11	14
Portugal	82	21	4	2	4	9	9	10	10	14
Qatar	150	33	4	3	11	16	22	13	16	29
Romania	82	19	4	2	4	8	9	9	11	17
Rwanda	118	30	6	5	5	9	11	12	20	23
Saudi Arabia	155	33	4	3	12	16	25	13	17	29
Senegal	132	33	7	5	6	9	12	16	22	29
Sierra Leone	126	32	7	6	6	9	13	16	19	25
Singapore	150	36	7	3	5	16	14	13	20	33
Slovak Republic	83	19	3	2	4	9	9	10	11	17
Slovenia	86	22	4	2	4	9	9	9	11	16
South Africa	62	15	2	3	2	7	8	14	14	4
Spain	84	22	4	2	4	9	9	7	11	14
Sri Lanka	131	31	7	4	8	16	13	8	10	26
Sudan	75	16	3	3	3	9	7	9	13	12
Sweden	70	17	4	2	4	7	7	8	10	12
Switzerland	79	22	3	2	3	9	9	10	10	12
São Tomé & Principe	108	28	6	5	6	9	13	9	15	17
Tajikistan	156	41	8	6	8	15	16	24	24	23

	All foods	Starchy staples	Oils, fats	Sugar	Beans, nuts, seeds	Vege-tables	Fruit	Dairy	Red meat	Poultry & fish
Tanzania	132	32	7	6	6	9	13	16	22	28
Thailand	140	32	6	4	5	15	13	11	19	31
Togo	73	24	5	1	3	2	4	11	17	15
Trinidad & Tobago	97	25	4	2	6	11	11	10	14	13
Tunisia	89	20	2	3	6	9	7	14	14	19
Turkey	79	20	4	2	4	9	9	8	6	16
Uganda	124	33	6	6	6	9	12	16	22	21
United Arab Emirates	152	29	3	3	12	16	25	12	19	29
United Kingdom	83	22	4	2	3	9	9	9	10	15
United States	62	13	3	1	3	6	7	8	11	12
Uruguay	57	14	3	1	5	7	5	6	7	8
Vietnam	161	39	6	4	6	15	15	10	21	40
Zambia	114	29	4	5	5	9	13	16	21	19
Zimbabwe	126	32	6	6	6	9	13	16	22	23
Mean	106	26	5	3	5	10	11	12	15	19

Source: Authors' calculations from the study sample. See Data and Methods for information on sample selection.

Table D2. Medians of country-level shares of food items priced relative to the full global list of ICP foods, by region and income level

	All foods*	Staples	Oils	Sugars	Legumes, nuts	Vegetables	Fruit	Dairy	Meat	Poultry, Eggs, Fish
Europe, US & Central Asia	40%	37%	44%	25%	33%	38%	36%	31%	33%	34%
Latin America	33%	29%	33%	25%	25%	38%	36%	28%	27%	22%
Middle East	65%	50%	44%	38%	79%	60%	88%	45%	52%	53%
East Asia & Pacific	68%	61%	67%	38%	42%	63%	52%	34%	58%	73%
South Asia	64%	67%	78%	50%	75%	58%	52%	31%	30%	59%
Sub-Saharan Africa	58%	53%	67%	63%	50%	38%	52%	53%	64%	52%
High income	39%	42%	26%	45%	44%	43%	33%	35%	38%	40%
Upper middle income	44%	46%	38%	50%	46%	47%	40%	43%	41%	41%
Lower middle income	54%	46%	50%	59%	57%	49%	46%	56%	52%	48%
Low income	54%	38%	61%	58%	67%	47%	51%	59%	52%	46%
Total	46%	43%	41%	52%	51%	46%	41%	46%	45%	43%

Notes. Results estimated by the authors for 141 countries from 2017 ICP national average prices as described in World Bank (2020) our Data and Methods section. *Refers to the number of food items priced in each country relative to the country with the longest food list, which was India, with 208 items.

Figure D2. Local polynomial plots of country-level ELD food group costs against the share of food group items priced in each country

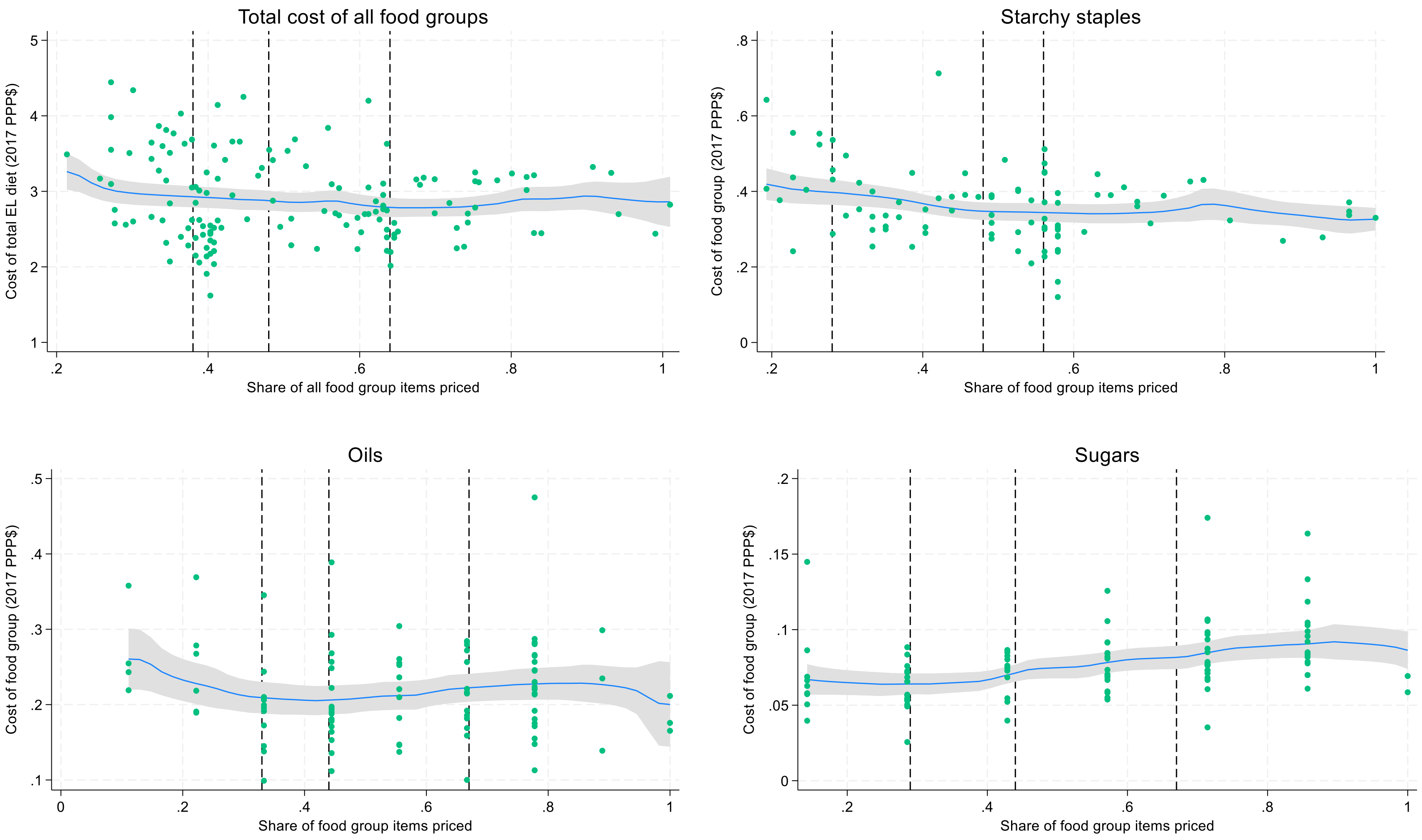

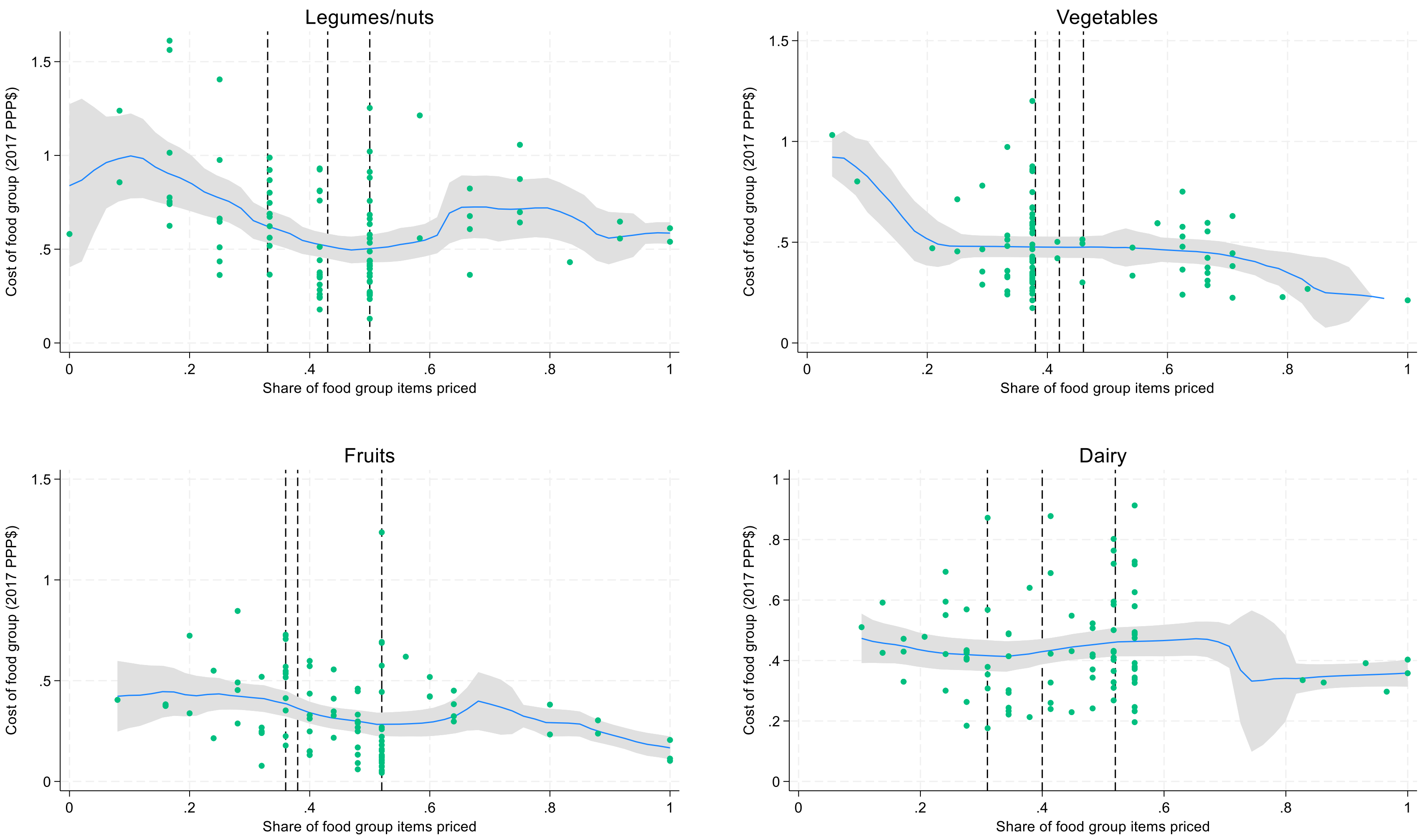
Legumes/nuts
Vegetables
Fruits
Dairy
Cost of food group (2017 PPP$)
Share of food group items priced

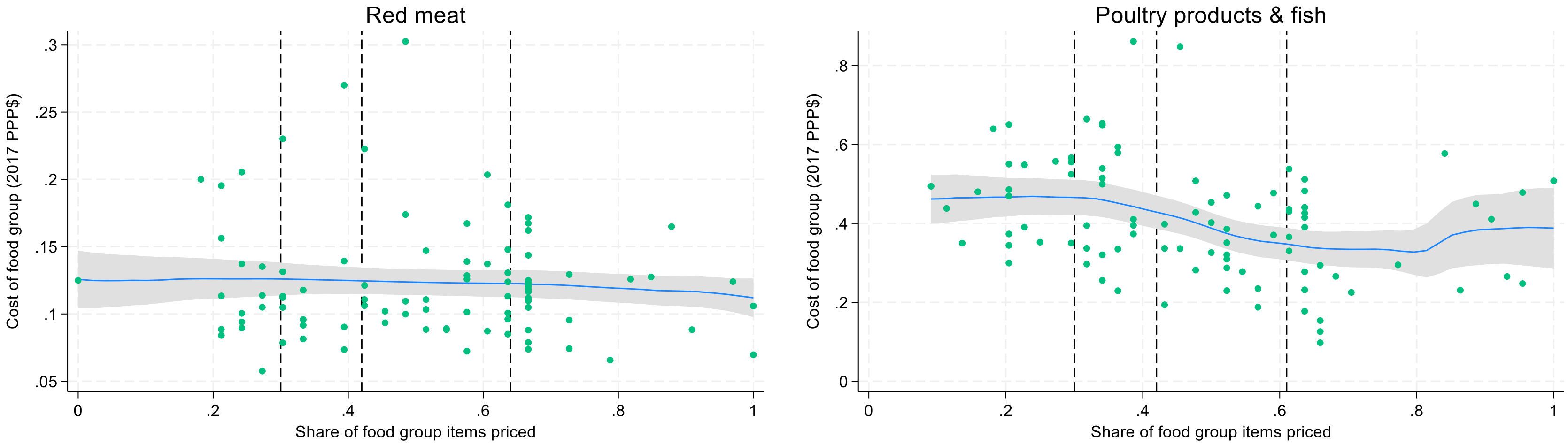

Notes: Solid red lines refer to percentiles of the x-axis variable. ELD Costs are residuals after netting out confounding factors. Grey lines represent 95% confidence intervals.

Table D3. Summary statistics of country-levels shares of food items priced relative to the full global list of ICP food

Panel A: Absolute number of food items priced for all foods and ELD food group

	**All foods**	**Starchy staples**	**Oils**	**Sugars**	**Legumes, nuts**	**Vegetables**	**Fruits**	**Dairy**	**Red meat**	**Poultry, eggs, fish**
Minimum	44	11	1	1	0	1	2	3	0	4
p25	79	20	4	2	4	9	9	9	10	13
p50	96	24	4	3	5	9	10	10	14	16
p75	131	32	6	5	6	11	13	15	21	27
Maximum	208	57	9	7	12	24	25	29	33	44

Panel B: Share of food items priced relative to total number of food items in each ELD food group

	**All foods***	**Starchy staples**	**Oils**	**Sugars**	**Legumes, nuts**	**Vegetables**	**Fruits**	**Dairy**	**Red meat**	**Poultry, eggs, fish**
Minimum	21%	19%	11%	14%	0%	4%	8%	10%	0%	9%
p25	38%	35%	39%	29%	33%	38%	36%	31%	30%	30%
p50	48%	44%	44%	43%	42%	38%	40%	34%	42%	39%
p75	64%	56%	67%	71%	50%	46%	52%	52%	64%	61%
Maximum	100%	100%	100%	100%	100%	100%	100%	100%	100%	100%

Notes. Results estimated by the authors for 141 countries from 2017 ICP national average prices as described in World Bank (2020) and in the main text. *Refers to the number of food items priced in each country relative to the country with the longest food list, which was India, with 208 items.

Table D4. Gauging the potential extent of urban bias in ICP national price surveys

	Number of countries	Countries covering rural & urban areas	Shops in rural areas if surveyed	Estimated rural population share	Nominal extent of urban bias	Rural coverage rating
Europe, US & Central Asia	41	0%	0%	31%	31%	Poor
Latin America & Caribbean	20	0%	0%	30%	30%	Poor
Middle East & North Africa	15	27%	3%	19%	16%	Average
East Asia & Pacific	17	59%	14%	38%	29%	Good
South Asia	7	100%	25%	68%	43%	Good
Sub-Saharan Africa	41	56%	16%	58%	44%	Average
High income	47	0%	0%	21%	21%	Poor
Upper middle income	36	28%	7%	35%	31%	Poor
Lower middle income	34	65%	16%	53%	40%	Average
Low income	24	50%	15%	66%	52%	Average
Total	141	31%	11%	40%	33%	

Notes: Authors' construction from ICP meta data, while the estimated rural population share is from the World Development Indicators (World Bank, 2019).

Figure D3. Partial local polynomial plots of country-level ELD food group costs against the extent of urban bias in food surveys

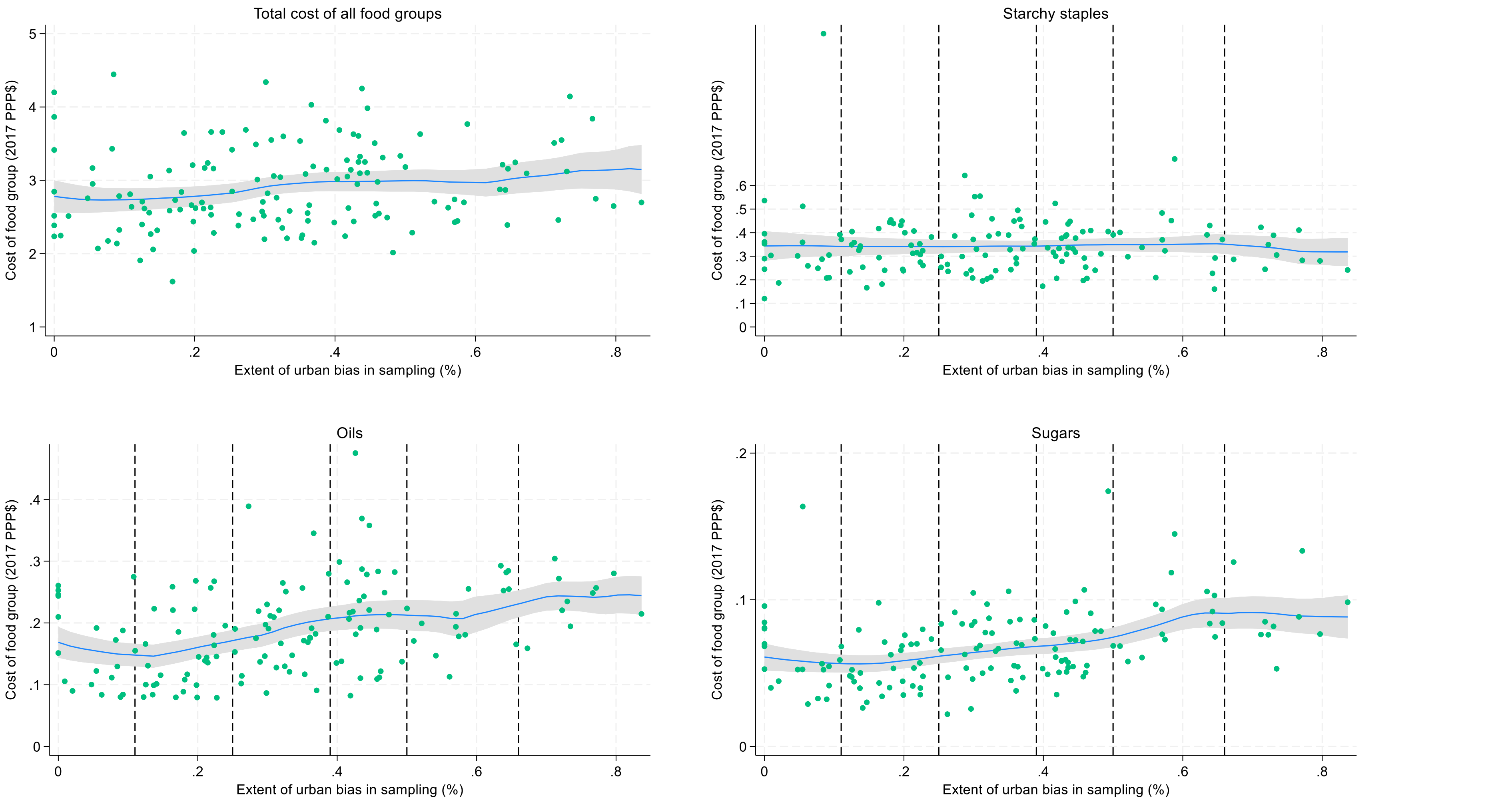

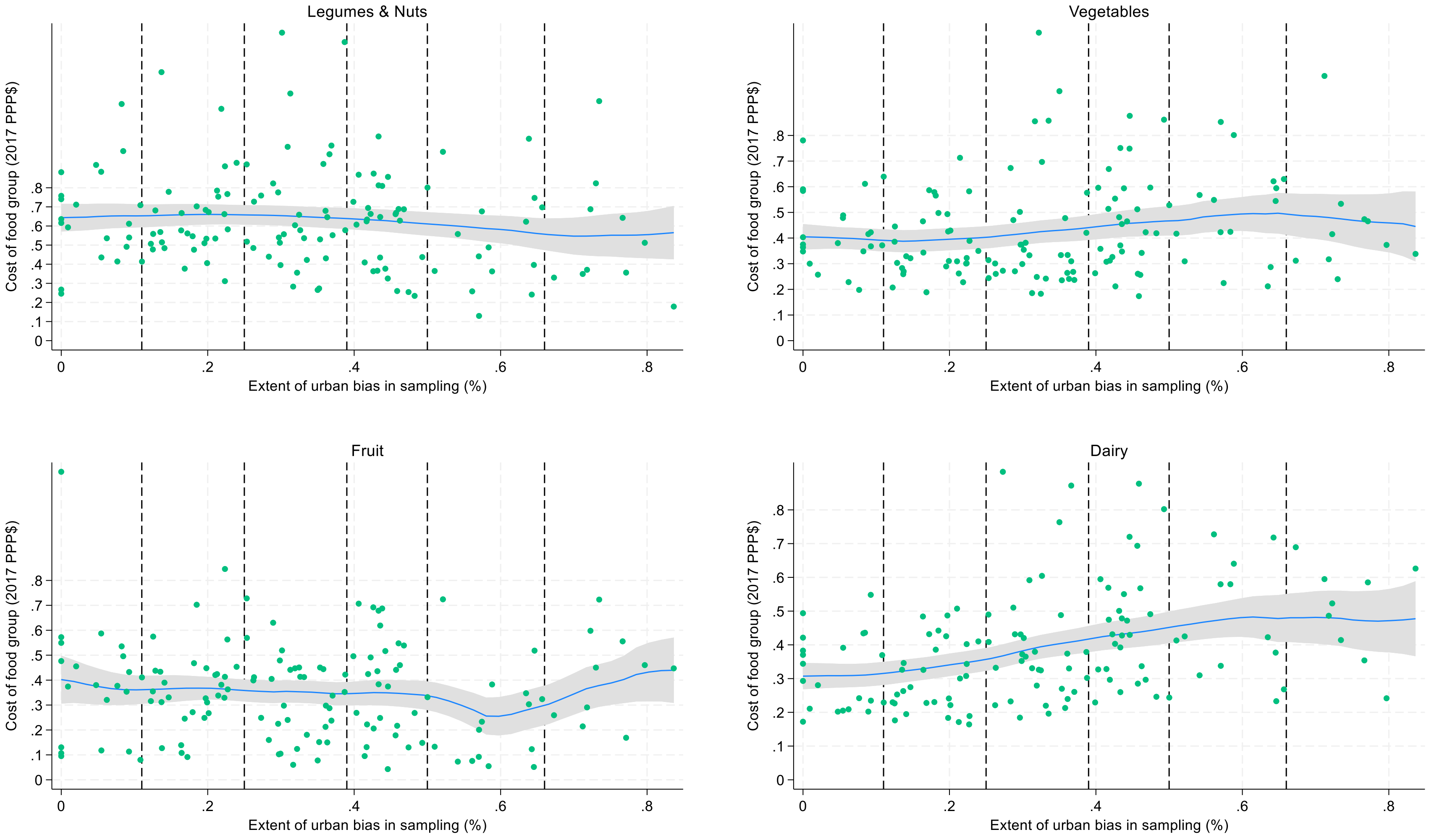
Legumes & Nuts
Vegetables
Fruit
Dairy
Cost of food group (2017 PPP$)
Extent of urban bias in sampling (%)
0
.1
.2
.3
.4
.5
.6
.7
.8

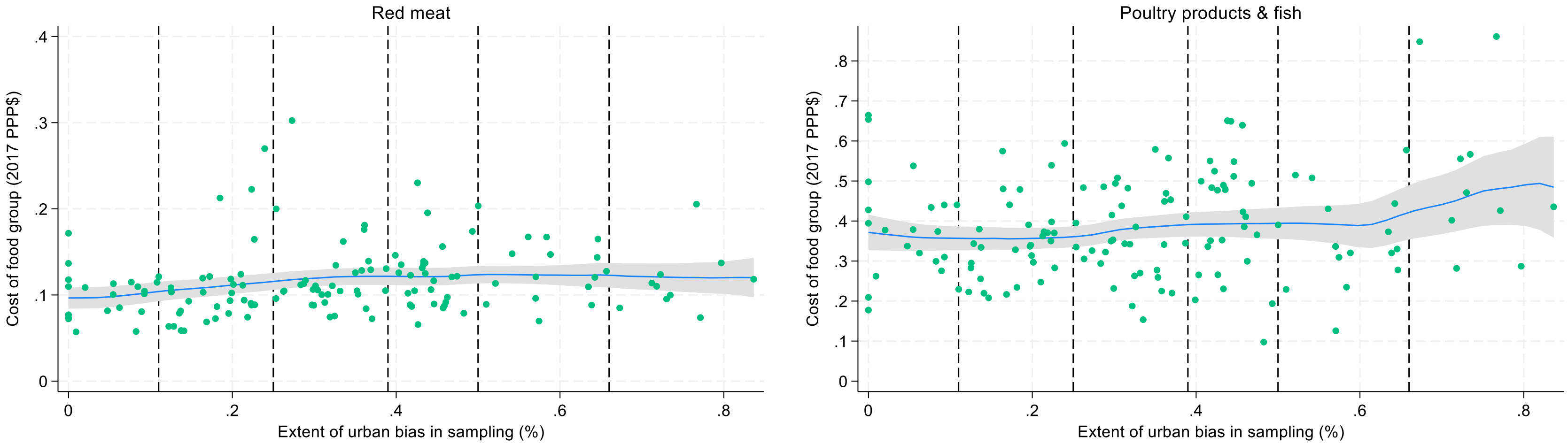

Notes: Solid red lines refer to x-axis percentiles. Plots are derived after netting out confounding factors. See the main test for further details. N=101 (excludes HICs).

Table D5. Robust regressions of ELD food group costs against "bias" measures in 141 countries, with additional controls

	Cost of starchy staples ($)	Cost of oils ($)	Cost of sugars ($)	Cost of legumes, nuts ($)	Cost of vegetables ($)	Cost of Fruit ($)	Cost of dairy ($)	Cost of red meat ($)	Cost of poultry & fish ($)
Product coverage [a] (1st spline, below knot)	-1.15*** (0.22)	-0.06 (0.04)	0.05** (0.02)	-0.40** (0.16)	-1.57*** (0.35)	-0.19 (0.16)	-0.81*** (0.23)	0.01 (0.02)	-0.33*** (0.09)
Product coverage [a] (2nd spline, above knot)	0.11** (0.05)	0.06 (0.08)	0.00 (0.01)	0.17 (0.15)	0.07 (0.10)	-0.36 (0.23)	-0.10 (0.09)	-0.02 (0.04)	0.30* (0.16)
Gap in rural survey coverage ("urban bias")	-0.05 (0.04)	0.01 (0.03)	0.00 (0.01)	0.07 (0.10)	-0.07 (0.08)	0.28*** (0.09)	0.12** (0.06)	0.02 (0.01)	0.05 (0.07)
Log GDP per capita	0.01 (0.01)	-0.02*** (0.01)	-0.01*** (0.00)	0.13*** (0.02)	-0.00 (0.02)	0.11*** (0.02)	-0.07*** (0.01)	-0.00 (0.00)	-0.00 (0.02)
Log rainfall	0.00 (0.01)	-0.02*** (0.00)	0.00 (0.00)	-0.01 (0.02)	0.04*** (0.01)	-0.01 (0.02)	-0.05*** (0.01)	0.01*** (0.00)	-0.03** (0.01)
Log temperature	0.02 (0.01)	0.01 (0.01)	-0.00 (0.00)	-0.10*** (0.03)	0.08*** (0.02)	-0.02 (0.03)	-0.01 (0.02)	0.01** (0.00)	0.01 (0.02)
Log transport logistics	-0.05*** (0.02)	-0.03*** (0.01)	-0.00 (0.00)	-0.21*** (0.04)	-0.05 (0.03)	-0.08** (0.03)	-0.00 (0.02)	-0.01 (0.01)	-0.06** (0.02)
Minimum product coverage (share)	19%	11%	14%	0%	4%	8%	10%	0%	9%
Product coverage turning point (percentile)	25th	75th	50th	75th	10th	50th	50th	75th	75th
Product coverage turning point (share of all ICP foods)	35%	66%	43%	50%	33%	52%	34%	61%	64%
Cost change from minimum share to turning point	-$0.18	N/A	N/A	-$0.20	-$0.46	N/A	-$0.19	N/A	-$0.18
R-squared	0.33	0.50	0.55	0.36	0.36	0.34	0.43	0.24	0.16

Notes: Results estimated by the authors for 93 LMICs from 2017 ICP national average prices as described in World Bank (2020) and in the text. These are robust regressions with standard errors in parentheses, and significant levels at the 10% (*), 5% (**) and 1% (***). [a] Product coverage is estimated with a spline regression with the knot (turning point) specific to each food group listed as a percentile of the distribution of the share of the global ICP food list, based on Figure D2.

Adjusting ELD costs for low product coverage

To assess the potential influence of product coverage bias on CoAHD estimates, we use the product coverage coefficients from Table D5 to adjust costs for any food group with a statistically significant coefficient, and then use those adjusted food group costs to aggregate up to an adjusted total ELD costs that is then used to re-calculate ELD affordability estimates (see the description in Appendix D). To adjust ELD costs, we limit how much an ELD food group cost can be adjusted downwards[1] by using the 25th percentile of the ELD cost distribution for each food group as a cost floor, though we acknowledge that this choice is arbitrary. One could, of course, set the floor as the minimum cost in the sample of countries, but such a cost could be an outlier because of measurement error, policy distortions, unusually high production potential, or other reasons. Moreover, we emphasize that this adjustment exercise is meant to be demonstrative; we are not claiming that these adjusted ELD costs are in any sense the 'true cost'.

Table D6 reports global and region-based comparisons of the baseline population-weighted ELD costs that are unadjusted and those that are adjusted to reduce potential bias from inadequate product coverage. Across all 137 countries, the total ELD cost only falls marginally, from $2.86 to $2.72 in 2017 PPP dollar terms, with half of this stemming from imposed reductions in the legumes/nuts food group costs for which product coverage is often quite poor. In specific regions the cost reductions are much larger than the global average. In Europe, the US, and Central Asia the ELD cost falls by 30 cents per day, in Latin America by 29 cents, in the Middle East and North Africa by 15 cents, in East Asia and the Pacific by 13 cents and in Sub-Saharan Africa by 11 cents. Only South Asia's costs are unchanged because of its already long food lists. While these adjustments sometimes appear modest, it is worth noting that 29 cents per day for a family of five – the Latin American example – amounts to around 40 PPP dollars per month, which is sizable.

[1] Indeed, it is possible that regression-based adjustments to ELD food group costs could result in negative costs.

Table D6. Population-weighted mean ELD and food group costs with and without adjustments for product coverage bias by World Bank regions (25th percentile floor method)

		Total ELD cost	Starchy staples	Oils	Sugars	Legumes, nuts	Vegetables	Fruits	Dairy	Red meat	Poultry & fish
All 141	Unadjusted	$2.86	$0.36	$0.18	$0.07	$0.61	$0.40	$0.35	$0.39	$0.12	$0.37
countries	Adjusted	$2.72	$0.34	$0.18	$0.07	$0.55	$0.39	$0.35	$0.38	$0.12	$0.35
Europe, US &	Unadjusted	$2.50	$0.31	$0.11	$0.05	$0.58	$0.40	$0.37	$0.27	$0.10	$0.32
Central Asia	Adjusted	$2.20	$0.25	$0.11	$0.05	$0.49	$0.30	$0.37	$0.25	$0.10	$0.28
Latin America	Unadjusted	$2.95	$0.38	$0.15	$0.06	$0.98	$0.34	$0.31	$0.32	$0.09	$0.32
	Adjusted	$2.66	$0.27	$0.15	$0.06	$0.88	$0.34	$0.31	$0.27	$0.09	$0.30
Middle East &	Unadjusted	$3.19	$0.37	$0.26	$0.07	$0.70	$0.33	$0.26	$0.57	$0.20	$0.42
North Africa	Adjusted	$3.04	$0.37	$0.26	$0.07	$0.59	$0.33	$0.26	$0.57	$0.20	$0.38
East Asia &	Unadjusted	$2.94	$0.41	$0.16	$0.07	$0.62	$0.43	$0.42	$0.43	$0.13	$0.28
Pacific	Adjusted	$2.81	$0.40	$0.16	$0.07	$0.52	$0.42	$0.42	$0.41	$0.13	$0.27
South Asia	Unadjusted	$2.87	$0.33	$0.22	$0.07	$0.56	$0.38	$0.34	$0.36	$0.12	$0.50
	Adjusted	$2.87	$0.33	$0.22	$0.07	$0.56	$0.38	$0.34	$0.36	$0.12	$0.50
Sub-Saharan	Unadjusted	$2.86	$0.36	$0.23	$0.10	$0.49	$0.47	$0.22	$0.47	$0.12	$0.40
Africa	Adjusted	$2.75	$0.33	$0.23	$0.10	$0.47	$0.45	$0.22	$0.46	$0.12	$0.38

Notes: Results estimated by the authors for 141 countries from 2017 ICP national average prices as described in in the text. Cost components are adjusted downwards using the spline regression coefficients, but to a floor equal to the median cost for each food group.

Appendix E. Varying physical activity levels and calorie requirements after applying a sedentary lifestyle assumption

Table E1. Revised estimates of the number and share of the population in 137 countries that are poor according to an EAT-*Lancet* reference diet food poverty line and country-specific non-food poverty lines assuming sedentary activity levels by country income level and geographic region

		1. New benchmark (column 2 of Table 4)		2. Assuming sedentary activity level for all		3. Difference (2.-1.)	
	**N**	**ELD poor (millions)**	**Headcount (%)**	**ELD poor (millions)**	**Headcount (%)**	**ELD poor (millions)**	**Headcount (%-points)**
**Global:**	**137**	**2,127.5**	**30.7**	**1,751.7**	**25.3**	**-375.8**	**-5.4**
**By income level:**							
High income	40	20.7	1.9	19.2	1.7	-1.5	-0.2
Upper middle income	36	294.6	12.1	219.8	9.1	-74.8	-3.0
Lower middle income	37	1,391.5	49.2	1,113.8	39.3	-277.7	-9.9
Low income	24	420.6	76.0	398.9	72.1	-21.7	-3.9
**By geographic region:**							
East Asia and Pacific	13	327.8	14.8	243.9	11.0	-83.9	-3.8
Europe, US & Central Asia	44	48.5	5.9	41.0	4.9	-7.5	-1.0
Latin America and Caribbean	18	89.8	17.1	75.4	14.3	-14.4	-2.8
Middle East and North Africa	10	72.0	28.9	56.3	22.6	-15.7	-6.3
North America	2	6.8	1.9	6.8	1.9	0.0	0.0
South Asia	7	882.0	51.2	670.9	39.0	-211.1	-12.2
Sub-Saharan Africa	43	700.6	68.7	657.5	64.5	-43.1	-4.2

Note: This table reports the number and share of people that cannot afford the EAT-*Lancet* reference diet (ELD) while factoring in a non-food cost component as well as adjustments. All estimates are based on price data from the 2017 International Comparison Program (ICP) database and factor in a country specific ELD costs and country specific non-food cost component based on an extension of the approach described in Allen (2017). They also adjust for differences in demographic profiles across countries and potential urban and product coverage biases in 2017 ICP data. Column 2 considers a scenario where all people follow a ‘sedentary’ lifestyle requiring minimal energy needs and downweighs the energy requirements in the ELD accordingly.
